# Hepatocyte Mettl3 Deficiency Drives Primary Sclerosing Cholangitis and Liver Fibrosis via Cholangiocyte‐Macrophage Crosstalk

**DOI:** 10.1002/advs.202512799

**Published:** 2025-12-22

**Authors:** Wenting Pan, Yuting Yong, Yuanshuai Li, Gaona Shi, Min Zhang, Lingfei Wan, Yue Zhao, Wenling Zhan, Yanli Lin, Qiaozhen Qin, Xupeng Chen, Yanli Ni, Haixu Chen, Wenzai Shi, Xiaomeng Guo, Juan Chen, Shuchen Liu, Youliang Wang, Bing Liu, Xinlong Yan

**Affiliations:** ^1^ Beijing Key Laboratory of Environmental and Viral Oncology College of Chemistry and Life Science Beijing University of Technology Beijing China; ^2^ State Key Laboratory of Experimental Hematology Institute of Hematology Senior Department of Hematology Fifth Medical Center Chinese PLA General Hospital Beijing China; ^3^ State Key Laboratory of Bioactive Substance and Function of Natural Medicines Institute of Materia Medica Chinese Academy of Medical Sciences & Peking Union Medical College Beijing China; ^4^ Department of Nephrology First Medical Center Chinese PLA General Hospital Beijing China; ^5^ Laboratory of Advanced Biotechnology Beijing Institute of Biotechnology Beijing China; ^6^ Institute of Geriatrics &National Clinical Research Center of Geriatrics Disease the Second Medical Center Chinese PLA General Hospital Beijing China; ^7^ Department of Hepatobiliary Surgery Peking University International Hospital Beijing China; ^8^ Key Laboratory of Precision Nutrition and Food Quality Department of Nutrition and Health China Agricultural University Beijing China; ^9^ Beijing Institute of Radiation Medicine Beijing China

**Keywords:** liver fibrosis, Mettl3, primary sclerosing cholangitis, Spp1^+^ cholangiocyte, Trem2^+^ macrophage

## Abstract

Effective therapies for primary sclerosing cholangitis (PSC), a progressive cholestatic liver disease characterized by biliary inflammation and fibrotic damage, remain limited due to an incomplete elucidation of its underlying molecular mechanisms. Although N6‐methyladenosine (m6A) RNA methylation has been implicated in hepatic pathophysiology, its role in PSC remains undefined. Here, we demonstrate that hepatocyte‐specific deletion of *Mettl3*, a critical m6A methyltransferase, induces spontaneous PSC‐like pathology characterized by ductular reaction and peribiliary fibrosis. Therapeutic restoration of *Mettl3* through genetic knock‐in or AAV8‐mediated hepatocyte‐specific overexpression significantly attenuated 3,5‐diethoxycarbonyl‐1,4‐dihydrocollidine (DDC)‐induced PSC progression. Integrated single‐cell and bulk transcriptomic profiles revealed an expansion of Trem2^+^ macrophages that interact with Spp1^high^ cholangiocytes via the Cd44‐Spp1 axis. Genetic ablation of *Trem2* or cholangiocyte‐specific deletion of *Spp1* significantly suppressed DDC‐induced biliary injury. Mechanistically, *Mettl3*‐deficient hepatocytes secreted higher levels of macrophage‐recruiting cytokines (such as Mif and Csf1), facilitating the recruitment of Trem2^+^ macrophage, which subsequently activated cholangiocytes through Cd44‐Spp1 signaling, exacerbated biliary inflammation and fibrosis. Notably, pharmacological activation of Mettl3 in adult hepatocytes substantially mitigated PSC progression and liver fibrosis. Collectively, our findings establish hepatocyte *Mettl3* deficiency as a pivotal driver of PSC pathogenesis and highlight the therapeutic potential of targeting the m6A epitranscriptome in cholestatic liver diseases.

## Graphical abstract

Schematic illustration demonstrating that hepatic *Mettl3* depletion significantly elevates the secretion of Mif and Csf1. This elevation facilitates Trem2^+^ macrophage infiltration and triggers cholangiocyte remodeling through the Spp1‐Cd44 interaction, resulting in spontaneous PSC development in vivo. This pathogenic cascade is reversed by hepatic *Mettl3* knock‐in, AAV8‐mediated *Mettl3* overexpression, and pharmacological Mettl3 activation (MA3).

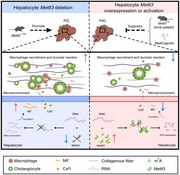

## 1 Introduction

Primary sclerosing cholangitis (PSC) is a progressive cholestatic liver disorder characterized by chronic biliary inflammation, periductal fibrosis, and stricture formation within the biliary tract, ultimately leading to liver cirrhosis and hepatic failure [1, 2]. Despite its clinical severity, therapeutic options remain limited to palliative care due to an insufficient understanding of its molecular mechanisms [3, 4]. To date, no effective treatments for PSC have been approved other than liver transplantation. Emerging evidence indicates that epitranscriptomic dysregulation contributes to cholestatic diseases [5, 6, 7]; however, the role of N6‐methyladenosine (m6A)—the most abundant internal RNA modification—in PSC pathogenesis remains largely unexplored [8].

The methyltransferase Mettl3, a key component of the m6A writer complex, displays context‐dependent roles in liver pathophysiology [9, 10]. While its oncogenic functions in hepatocellular carcinoma and intrahepatic cholangiocarcinoma are well‐documented [11, 12, 13, 14, 15, 16], Mettl3's influence on non‐neoplastic liver diseases exhibits notable spatiotemporal specificity [17]. Perinatal hepatocyte‐specific ablation of Mettl3 triggers fetal non‐alcoholic steatohepatitis (NASH) [10, 18] and exacerbates Acetaminophen (APAP)‐induced liver injury [19], whereas its deletion in adult hepatocytes results in no apparent phenotype [18, 20, 21]. Conversely, Mettl3 deficiency in hepatic stellate cells mitigates toxin‐induced fibrosis [22], underscoring its cell type‐ and developmental stage‐dependent regulatory complexity. Despite these findings, the role of Mettl3 in cholestatic injury—particularly its potential function in orchestrating immune‐ductular crosstalk in PSC— remains unexplored.

Here, we define hepatocyte‐specific *Mettl3* expression as a crucial regulator of biliary homeostasis. Hepatocyte‐specific *Mettl3* knockout mice (Alb‐Cre; *Mettl3* ^fl/fl^, hereinafter referred to as *Mettl3* ^∆Hep^) spontaneously develop PSC‐like features, including ductular reactions and peribiliary fibrosis, with exacerbated pathology observed in the DDC‐induced PSC model. Both hepatic *Mettl3* genetic knock‐in and AAV8 mediated *Mettl3* overexpression significantly attenuate biliary injury and fibrotic progression in the DDC‐challenged PSC model. Integrated single‐cell and bulk transcriptomic analyses uncover a pathogenic triad linking *Mettl3*‐deficient hepatocytes to Trem2^+^ macrophages and Spp1^high^ cholangiocytes. Notably, genetic ablation of *Trem2* or cholangiocyte‐specific *Spp1* deficiency attenuates DDC‐induced PSC. Mechanistically, hepatocyte *Mettl3* deficiency promotes m6A‐dependent Mif/Csf1 secretion, leading to Trem2^+^ macrophage recruitment and ultimately facilitating the Spp1‐Cd44 axis‐mediated interactions between Spp1^high^ cholangiocytes and Trem2^+^ macrophages. Importantly, pharmacological activation of Mettl3 effectively ameliorates PSC and liver fibrosis in vivo. Our findings thus establish Mettl3 as a central orchestrator of hepatocyte‐driven immune‐ductular crosstalk in PSC pathogenesis, thereby highlighting m6A modulation as a promising therapeutic strategy for cholestatic liver disease.

## 2 Results

### 2.1 Hepatocyte‐Specific Deletion of Mettl3 Induces Spontaneous PSC Pathogenesis

**FIGURE 1:**
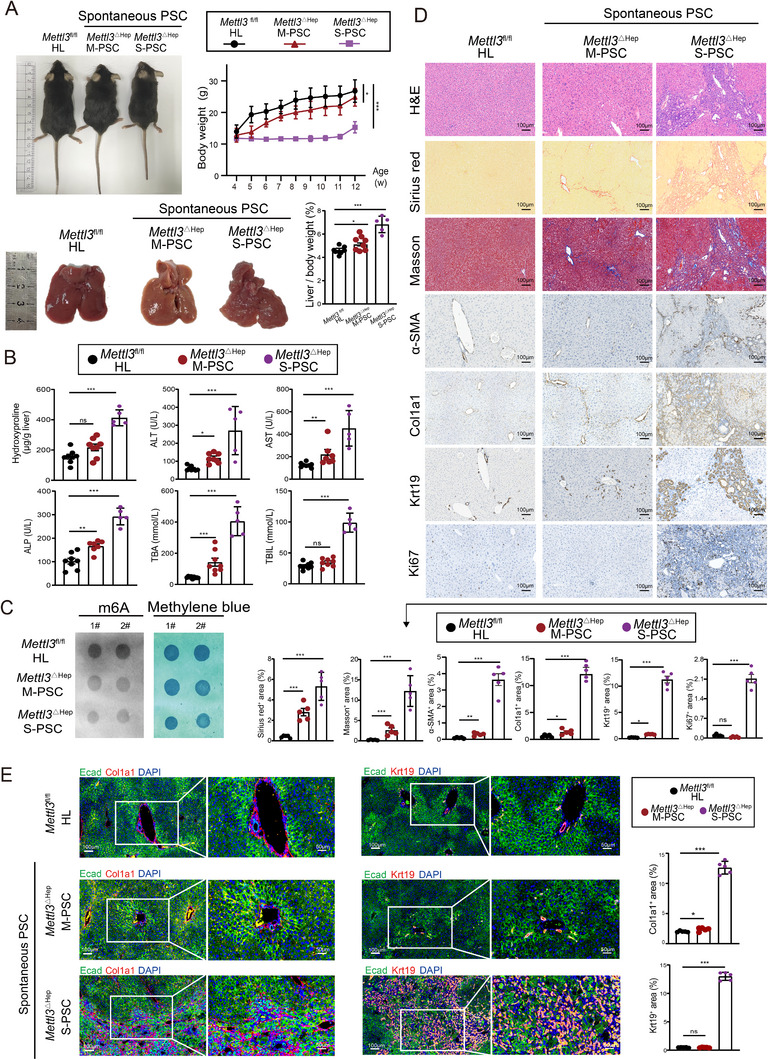
Hepatocyte‐specific *Mettl3* deficiency induces spontaneous PSC pathology. A) Representative images of longitudinal body weight analysis, gross liver morphology, and liver‐to‐body weight ratios of 12‐week‐old *Mettl3* ^ΔHep^ mice, displaying mild (*Mettl3* ^ΔHep^ M‐PSC, *n* = 8) or severe (*Mettl3* ^ΔHep^ S‐PSC, *n* = 5) phenotypes, compared to Mettl3 ^fl/fl^ controls (*n* = 8). Data are represented as mean± SEM. B) Hepatic hydroxyproline content and serum biochemical markers (ALT, AST, ALP, TBA, and TIBL) in *Mettl3* ^∆Hep^ mice versus *Mettl3* ^fl/fl^ controls. C) Dot blot analysis of m6A methylation levels in *Mettl3* ^∆Hep^ mice versus *Mettl3* ^fl/fl^ controls, with Methylene blue staining serving as an RNA loading control. D) Representative images of histopathological assessment using H&E, Sirius Red, Masson's trichrome staining, and immunohistochemical (IHC) staining of α‐SMA, Col1a1, Krt19, and Ki67, with quantification of the positive area. Scale bars: 100 µm. E) Representative images of multiplex immunofluorescence staining of Ecad (green), Col1a1 (red), Krt19 (red), and DAPI (blue) in *Mettl3* ^∆Hep^ mice versus *Mettl3* ^fl/fl^ controls. Scale bars: 100 µm (main panels), 50 µm (inserts). Data represent mean ± SEM; *p < 0.05, **p < 0.01, ***p < 0.001 by two‐tailed unpaired Student's t‐test.

To investigate the role of Mettl3 in PSC progression, we generated hepatocyte specific *Mettl3* knockout mice (*Mettl3* ^△Hep^) by crossing *Mettl3* ^fl/fl^ mice with Alb‐Cre transgenic mice (Figure S1A). Ludwig staging revealed that 83.02% of *Mettl3* ^△Hep^ mice (*n* = 53) spontaneously developed mild PSC (M‐PSC, Stage I, *n* = 44), while 16.98% progressed to severe PSC (S‐PSC, Stage II, *n* = 9) (Figure S1B), with normal *Mettl3* ^fl/fl^ mice serving as healthy liver controls (HL, *n* = 20). *Mettl3* ^△Hep^ mice exhibited reduced body weights, enhanced liver‐to‐body weight ratios (Figure 1A), elevated serum biomarkers of cholestatic injury, including Hydroxyproline, alanine aminotransferase (ALT), aspartate aminotransferase (AST), alkaline phosphatase (ALP), total bile acids (TBA), and total bilirubin (TBIL) (Figure 1B), accompanied by diminished hepatic m6A levels (Figure 1C). Histopathological analysis revealed extensive collagen deposition (Sirius Red staining, Masson's trichrome staining, activation of Col1a1^+^ and α‐SMA^+^ myofibroblasts), and enhanced proliferation and expansion of Krt19^+^ cholangiocytes in both M‐PSC and S‐PSC *Mettl3* ^△Hep^ mice (Figure 1D). Liver fibrosis‐related markers were significantly upregulated at both mRNA and protein levels in *Mettl3* ^△Hep^ mice (Figure S1C,D). Notably, enhanced fibrosis and ductular reaction were marked by an increase in Col1a1‐ and Krt19‐positive areas (Figure 1E; Figure S1E). Immune dysregulation, characterized by F4/80^+^ macrophage infiltration and disrupted lobular architecture, further defined the inflammatory liver microenvironment in *Mettl3* ^△Hep^ PSC (Figure S1F,G). Collectively, hepatocyte‐specific deletion of *Mettl3* induces spontaneous PSC characterized by progressive fibrosis, ductular hyperplasia, and immune dysregulation.

### 2.2 Hepatocyte Mettl3 Deficiency Exacerbates Chemically Induced PSC and Liver Fibrosis

**FIGURE 2:**
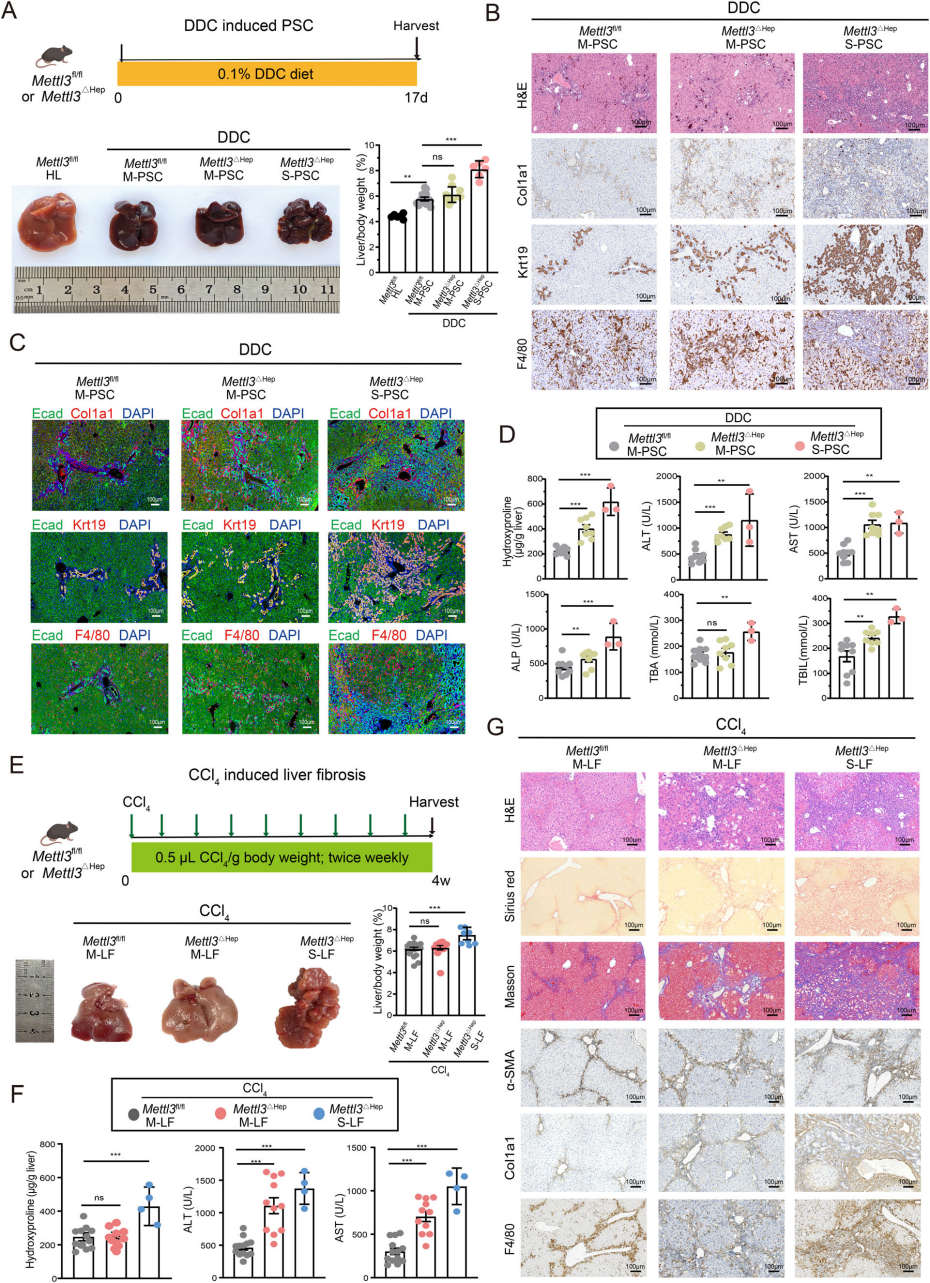
Hepatic *Mettl3* depletion exacerbates DDC‐induced PSC and liver fibrosis. A) Experimental design and liver morphology of DDC‐induced PSC models in *Mettl3* ^ΔHep^ (*Mettl3* ^ΔHep^ M‐PSC, *n* = 11; *Mettl3* ^ΔHep^ S‐PSC, *n* = 6), *Mettl3* ^fl/fl^ mice (*Mettl3* ^fl/fl^ M‐PSC, *n* = 12), and healthy livers of *Mettl3* ^fl/fl^ under standard diet conditions (*Mettl3* ^fl/fl^ HL, *n* = 6). B) Representative images of histopathology analysis of H&E, Sirius Red, and Masson's trichrome staining in *Mettl3* ^ΔHep^ versus *Mettl3* ^fl/fl^ mice of DDC‐induced PSC model. C) Representative images of multiplex immunofluorescence imaging of Ecad (green) with Krt19 (red), Col1a1 (red), F4/80 (red), and DAPI nuclear counterstain (blue). Scale bars: 100 µm. D) Hepatic hydroxyproline content and serum biomarkers, including ALT, AST, ALP, TBA, and TIBL, in the DDC‐induced PSC models of *Mettl3* ^ΔHep^ (*Mettl3* ^ΔHep^ M‐PSC, *n* = 8; *Mettl3* ^ΔHep^ S‐PSC, *n* = 3) versus *Mettl3* ^fl/fl^ mice (*Mettl3* ^fl/fl^ M‐PSC, *n* = 9). E) Experimental design and liver morphology of CCl_4_‐induced liver fibrosis model in *Mettl3* ^ΔHep^ (moderate liver fibrosis, *Mettl3* ^ΔHep^ M‐LF, *n* = 16; severe liver fibrosis, *Mettl3* ^ΔHep^ S‐LF, *n* = 8) vs. Mettl3 ^fl/fl^ controls (moderate liver fibrosis, *Mettl3* ^fl/fl^ M‐LF, *n* = 20). F) Hydroxyproline content and serum ALT/AST levels in *Mettl3* ^ΔHep^ (*Mettl3* ^ΔHep^ M‐LF, *n* = 11; *Mettl3* ^ΔHep^ S‐LF, *n* = 4) compared with *Mettl3* ^fl/fl^ (*Mettl3* ^fl/fl^ M‐LF, *n* = 13) in the CCl_4_‐induced liver fibrosis model. G) Representative images of histopathological evaluation using H&E, Sirius Red, Masson's trichrome staining, and immunohistochemical staining of α‐SMA, Col1a1, and F4/80. Scale bars: 100 µm. Data represent mean ± SEM; *p < 0.05, **p < 0.01, ***p < 0.001 by two‐tailed unpaired Student's t‐test.

The pathogenic effects of hepatocyte *Mettl3* loss were investigated in chemically induced PSC models. In DDC‐treated mice, *Mettl3* ^△Hep^ (*n* = 17) exhibited both M‐PSC (*n* = 11, 64.71%) and S‐PSC phenotypes (*n* = 6, 35.29%, Figure S2A), characterized by enhanced liver‐to‐body weight ratios (Figure 2A), pronounced ductular reaction (marked by elevated Krt7 and Krt19 expression), exacerbated fibrosis (increased staining of Sirius Red, Masson's trichrome, Col1a1, and α‐SMA), and increased macrophage infiltration (F4/80 accumulation) (Figure 2B; Figure S2B,C). Immunofluorescence staining revealed the co‐localization of hyperplastic cholangiocytes, enriched collogen, and macrophages within the regions of ductular injury (Figure 2C). Additionally, hydroxyproline content and serum biomarkers (ALT, AST, ALP, TBA, and TBIL) were elevated in the DDC‐induced PSC model of *Mettl3* ^△Hep^ mice (Figure 2D). Similar exacerbation was observed in the carbon tetrachloride (CCl_4_)‐induced liver fibrosis model, where *Mettl3* ^△Hep^ mice developed severe liver fibrosis, increased hydroxyproline and ALT/AST levels, extensive collagen deposition (Sirius Red and Masson's trichrome staining), elevated Col1a1 and α‐SMA expression, and expanded cholangiocyte and macrophage populations (Figure 2E–G; Figure S2D–G). Collectively, these findings establish hepatocyte *Mettl3* as a critical suppressor of injury‐driven cholangiopathy and liver fibrosis.

To determine whether adult hepatocyte‐specific deletion of *Mettl3* affects PSC progression, we administered AAV8‐TBG‐cre virus into 6‐8‐week‐old *Mettl3* ^fl/fl^ mice. In contrast to the marked PSC phenotype observed in constitutive *Mettl3* ^△Hep^ mice, the AAV8‐TBG‐cre group showed no difference in PSC process compared to AAV8 mock group (Figure S3). This phenotype observed in PSC is consistent with prior studies showing that adult hepatocyte deletion of *Mettl3*, using either the Alb‐cre ERT2 [21] or AAV8‐TBG‐cre [20] system, does not induce overt liver pathology.

### 2.3 Mettl3 Overexpression Ameliorates PSC Progression and Liver Fibrosis

**FIGURE 3:**
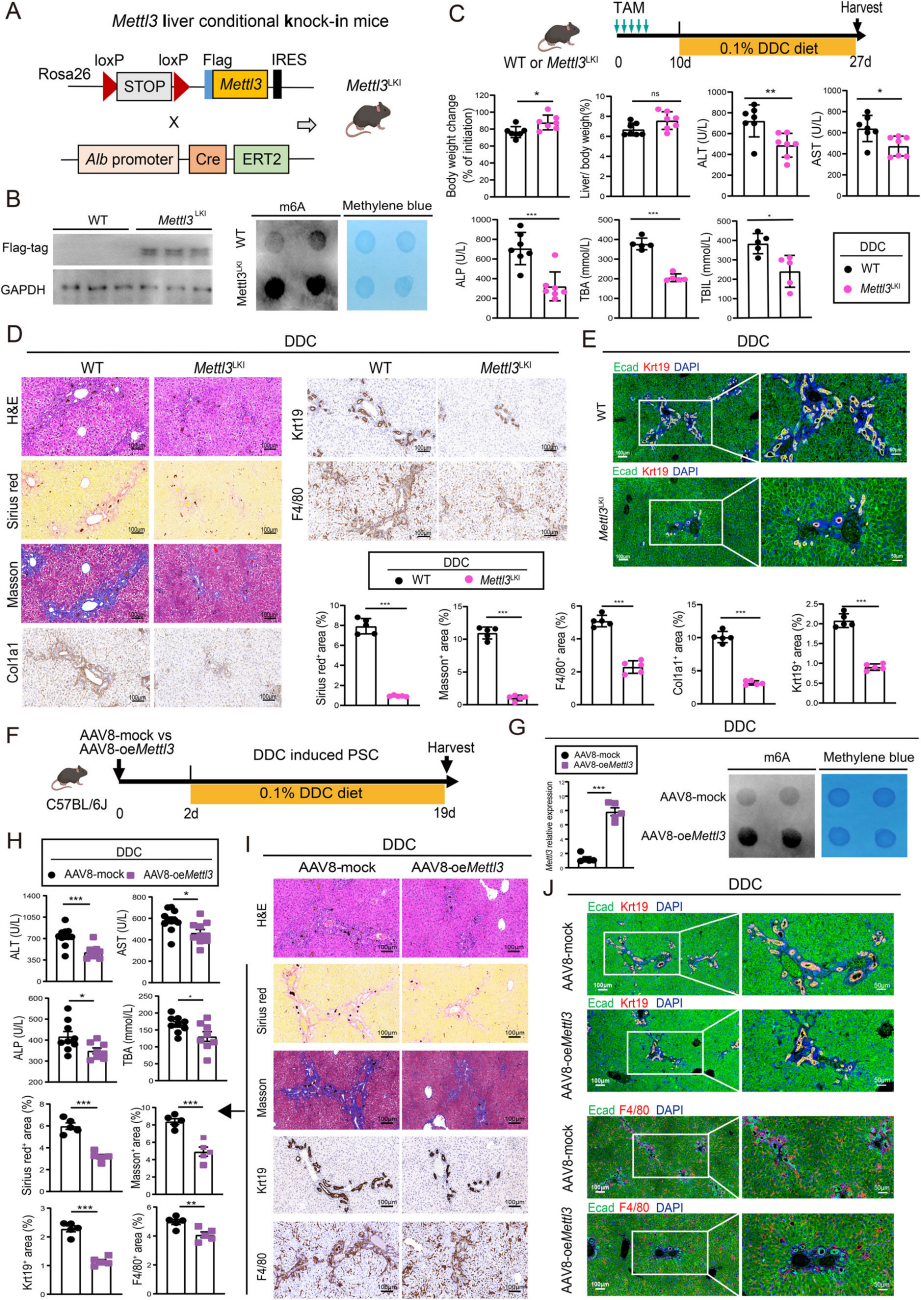
Both *Mettl3* knock‐in and AAV8‐mediated *Mettl3* overexpression ameliorate DDC‐induced PSC and liver fibrosis. A) Experimental scheme illustrating the design of induced hepatocyte‐specific *Mettl3* knock‐in mice (*Mettl3* ^LKI^). B) Western blot and m6A dot blot analysis confirmed Mettl3 overexpression and elevated m6A levels in *Mettl3* ^LKI^ mice compared to controls. C) Experimental design for *Mettl3* ^LKI^ and control mice subjected to the DDC‐induced PSC model. Comparison of body weight changes, liver‐to‐body weight ratios, and serum ALT, AST, ALP, TBA, and TBIL levels of *Mettl3* ^LKI^ mice (*n* = 7) versus control mice (*n* = 7) following DDC‐induced PSC. D) Representative images of histopathology examination using H&E, Sirius Red, and Masson's trichrome staining, IHC staining of Col1a1, Krt19, and F4/80 in *Mettl3* ^LKI^ mice versus control mice of DDC‐induced PSC. The percentage of positive area was quantified. E) Representative images of multiplex immunofluorescence analysis of Ecad (green), Krt19 (red), and DAPI (blue) of *Mettl3* ^LKI^ compared to control mice in the DDC‐induced PSC model. F) Experimental design depicting AAV8‐mediated *Mettl3* overexpression (AAV8‐oe*Mettl3*) in the DDC‐ induced PSC model. G) *Mettl3* expression and global m6A methylation levels in liver samples from DDC‐induced PSC mice treated with AAV8‐oeMettl3 or AAV8‐mock control, as measured by RT‐qPCR and m6A dot blot analysis, respectively. H) Serum levels of ALT, AST, ALP, and TBA in AAV8‐oe*Mettl3* (*n* = 10) compared to AAV8‐mock mice (*n* = 11) in DDC‐induced PSC. I) Representative images of histopathology analysis of H&E, Sirius Red, and Masson's trichrome staining, IHC staining for Krt19 and F4/80, with quantification of the positive area. J) Representative images of immunofluorescence staining analysis of Ecad (green), Krt19 (red), and F4/80 (red) of AAV8‐oe*Mettl3* versus AAV8‐mock mice in the DDC‐induced PSC model. Scale bars: 100 µm. Data represent mean ± SEM; *p < 0.05, **p < 0.01, ***p < 0.001 by two‐tailed unpaired Student's t‐test.

To characterize the contribution of ectopic Mettl3 expression in PSC progression, we employed two approaches: hepatocyte‐specific *Mettl3* knock‐in and AAV8‐mediated hepatic *Mettl3* overexpression. To generate hepatocyte‐specific *Mettl3* knock‐in mice (*Mettl3* ^LKI^), we crossed Rosa26‐lsl‐*Mettl3* mice, which harbor a loxP‐flanked full‐length *Mettl3 cDNA* in the Rosa26 locus, with Alb‐CreERT2 mice (Figure 3A) [11]. Western blot and dot blot assays confirmed increased ectopic Mettl3 expression and elevated RNA m6A levels in tamoxifen (TAM)‐induced *Mettl3* ^LKI^ mice compared to controls (Figure 3B). Following TAM administration, *Mettl3* ^LKI^ mice and their wild‐type (WT) littermates were subjected to a DDC diet to induce PSC. *Mettl3* ^LKI^ mice exhibited significantly increased body weight gain, but no difference in liver‐to‐body weight ratios, alongside reduced serum levels of ALT, AST, ALP, TBA, and TBIL (Figure 3C), indicating ameliorated PSC injury. Histological evaluation further demonstrated that *Mettl3* ^LKI^ mice were resistant to DDC‐induced PSC, as evidenced by decreased Sirius Red and Masson's trichrome staining, and fewer myofibroblasts (Col1a1) and cholangiocytes (Krt19), diminished F4/80^+^ macrophage infiltration (Figure 3D). The amelioration of PSC was further confirmed by immunofluorescence staining of Ecad and Krt19 (Figure 3E).

Consistent therapeutic efficacy of Mettl3 restoration was demonstrated through AAV8‐mediated *Mettl3* overexpression (AAV8‐oe*Mettl3*) in the DDC‐induced PSC model (Figure 3F). Administration of AAV8‐oe*Mettl3* increased m6A levels and *Mettl3* expression (Figure 3G), reduced Hydroxyproline and serum levels of ALT, AST, ALP, and TBA (Figure 3H; Figure S4A), and normalized ductular reactions (Krt19) as well as liver fibrosis markers (α‐SMA and Col1a1 staining) (Figure S4B). IHC analyses revealed decreased collagen deposition, ductular reaction, and macrophage infiltration in DDC‐induced PSC mice following AAV8‐oe*Mettl3* treatment (Figure 3I,J; Figure S4C). Similar therapeutic effects were observed in the CCl_4_‐induced liver fibrosis model, where AAV8‐mediated *Mettl3* overexpression suppressed liver fibrosis and inflammation (Figure S5A–E). Collectively, these findings indicate that both hepatocyte‐specific *Mettl3* knock‐in and AAV8‐mediated *Mettl3* overexpression in adult hepatocytes significantly ameliorated the progression of DDC‐induced PSC.

### 2.4 Single‐Cell RNA Sequencing Identify Trem2^+^ Macrophages as Pathogenic Drivers in Mettl3^△^^Hep^ PSC

**FIGURE 4:**
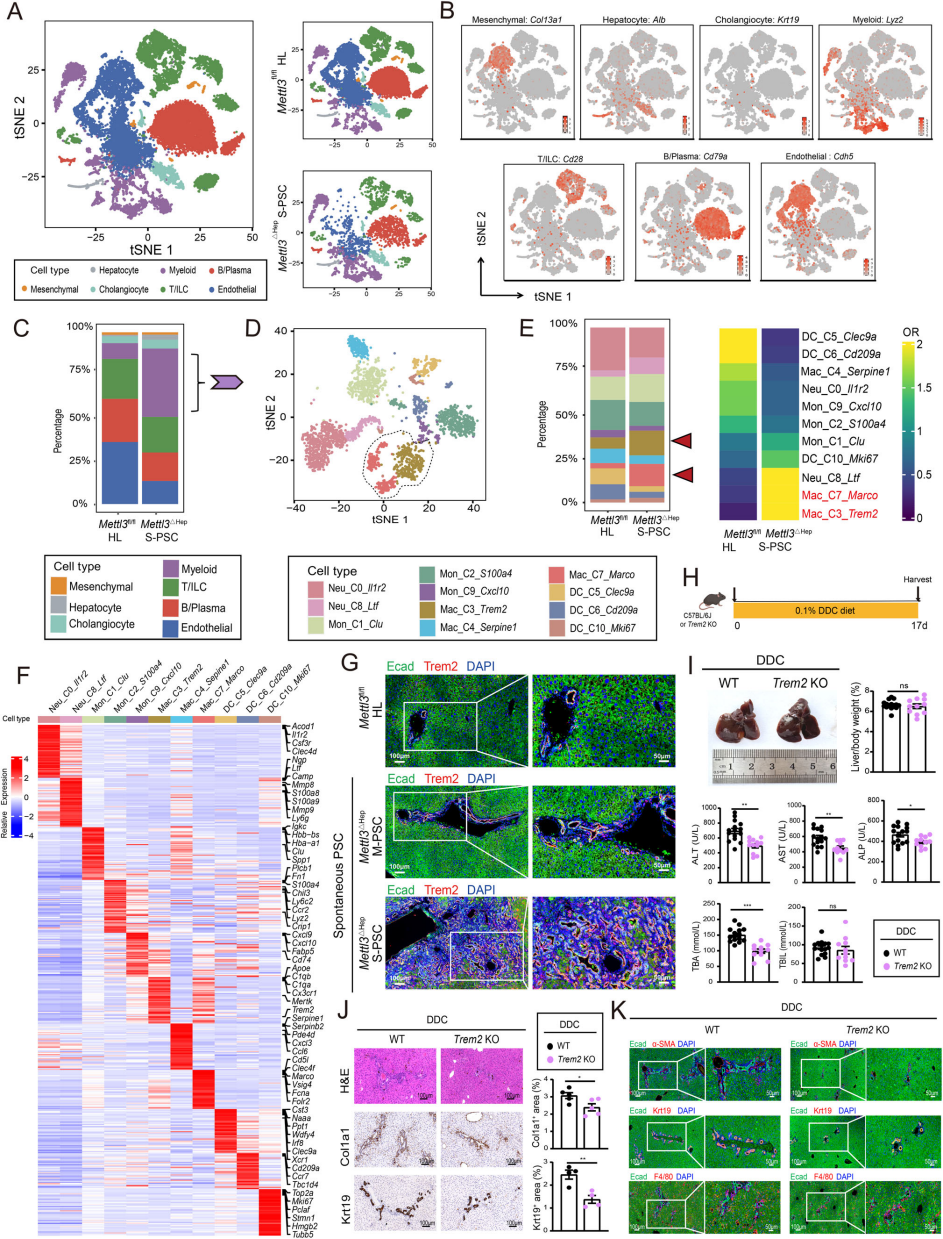
Single‐cell profiling and functional assays identify Trem2^+^ macrophages as pathogenic drivers in Mettl3^△Hep^ PSC. A) t‐SNE plots illustrating distinct major liver cell clusters (left) and cell distribution (right) in *Mettl3* ^ΔHep^ S‐PSC compared to *Mettl3* ^fl/fl ^controls, color‐coded by annotated cell types. B) t‐SNE visualization displaying canonical marker gene expression for major cell types. C) Composition analysis revealing significant enrichment of myeloid cells in *Mettl3* ^ΔHep^ S‐PSC versus *Mettl3* ^fl/fl ^controls (left). D) t‐SNE plots of myeloid subtypes (left) alongside stacked bar plots displaying the distribution of myeloid subtypes (right). E) Odds ratios (OR) were calculated to reflect the preferences of myeloid subtype distribution. F) Heatmap showing differentially expressed genes across myeloid subtypes. G) Representative images of immunofluorescence validation of Trem2^+^ macrophages (red) adjacent to Ecad^High^ cholangiocytes (green) in the livers of spontaneous *Mettl3* ^ΔHep^ M‐PSC, *Mettl3* ^ΔHep^ S‐PSC, and *Mettl3* ^fl/fl^ controls. H) Experimental scheme depicting the DDC‐induced PSC model in *Trem2* KO mice (*n* = 12) and WT controls (*n* = 15). I) Gross liver morphology, liver‐to‐body weight ratios, serum biomarkers, and representative images of histopathology analysis of the DDC‐induced PSC in *Trem2* KO and WT mice. J) Representative images of multiplex immunofluorescence analysis of Ecad (green), α‐SMA (red), and DAPI (blue); Ecad (green), Krt19 (red), and DAPI (blue); Ecad (green), F4/80 (red), and DAPI (blue) in the DDC‐induced PSC of *Trem2* KO mice versus WT controls. Scale bars: 100 µm. Data represent mean ± SEM; *p < 0.05, **p < 0.01, ***p < 0.001 by two‐tailed unpaired Student's t‐test.

Single‐cell RNA sequencing analysis comparing *Mettl3* ^△Hep^ S‐PSC livers with *Mettl3* ^fl/fl^ controls revealed extensive microenvironmental remodeling. Unsupervised clustering identified seven major cell types, highlighting a pronounced enrichment of myeloid cells in *Mettl3* ^△Hep^ livers and predominance of endothelial cells in control counterparts (Figure 4A–C; Figure S6A–C). High‐resolution sub‐clustering of myeloid cells further delineated 11 distinct subsets, with notable expansion of Trem2^+^ macrophages (Mac_C3_*Trem2*) and Marco^+^ macrophages (Mac_C7_*Marco*) in *Mettl3* ^△Hep^ S‐PSC livers (Figure 4D–F; Figure S7A–E). Multiplex immunofluorescence confirmed the increased presence of Trem2^+^ macrophage populations in *Mettl3* ^△Hep^ livers (Figure 4G). To functionally interrogate Trem2's role in PSC progression, *Trem2* knockout (KO) mice were subjected to DDC‐induced PSC (Figure 4H). Genetic ablation of *Trem2* significantly attenuated disease pathology, as demonstrated by reduced ductular reaction markers (Krt19 and Krt7), diminished fibrosis indicators (α‐SMA and Col1a1), and decreased macrophage infiltration (F4/80), compared to wild‐type (WT) controls (Figure 4I; Figure S8A,B). This protective effect was similarly observed in the CCl_4_‐induced hepatic fibrosis model, where *Trem2* KO mice exhibited reduced collagen deposition compared to *WT* counterparts (Figure S8C–E). Notably, *Marco* deficiency did not affect fibrotic progression in the CCl_4_‐induced fibrosis model (Figure S8F). Collectively, this comprehensive analysis identifies Trem2^+^ macrophages as critical mediators of microenvironmental reprogramming in *Mettl3* ^△Hep^‐driven cholangiopathy, highlighting a promising therapeutic target for PSC intervention.

### 2.5 The Spp1‐Cd44 Axis Mediates Cholangiocyte‐Macrophage Crosstalk in Mettl3^△^^Hep^ Driven PSC

**FIGURE 5:**
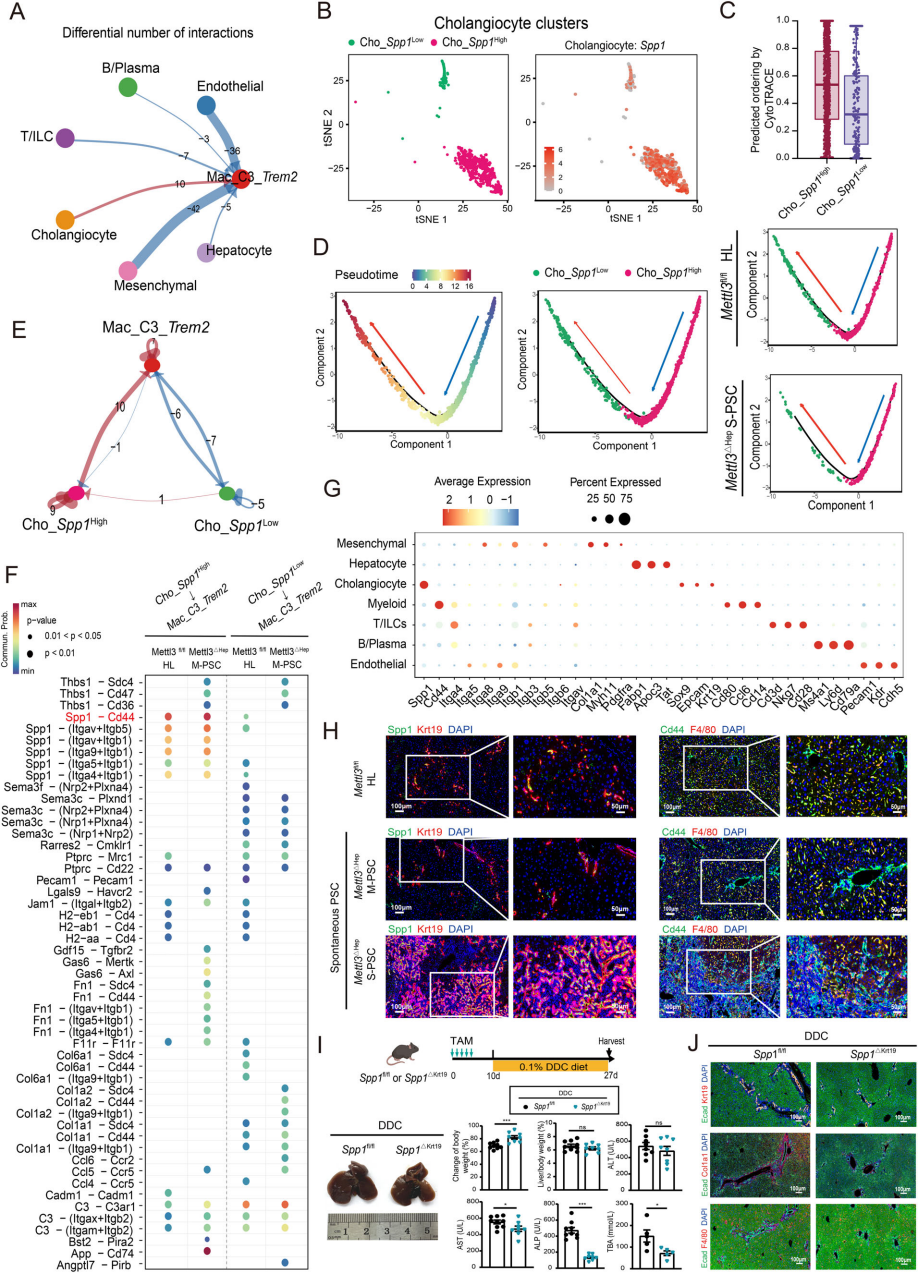
The Spp1‐Cd44 axis facilitates cholangiocyte‐macrophage crosstalk in the *Mettl3* ^ΔHep^ PSC. A) Circle plot illustrating distinct patterns of cellular interactions between Mac_C3_*Trem2* and other predominant cell types, in *Mettl3* ^ΔHep^ S‐PSC compared to *Mettl3* ^fl/fl ^controls. B) t‐SNE visualization of cholangiocyte subclusters in distinct colors (left) and Spp1 expression pattern (right). C) CytoTRACE analysis predicting developmental potential between Cho‐*Spp1* ^High^ and Cho‐*Spp1* ^Low^ cholangiocyte subclusters. D) Pseudotime analysis illustrating the developmental trajectory of cholangiocyte subtypes, highlighting enrichment of Cho‐*Spp1* ^high^ subsets in the *Mettl3* ^ΔHep^ S‐PSC mice. E) Differential communication network displaying the number of interactions between Mac‐C3‐*Trem2* and Cho‐*Spp1* ^High^ or Cho‐*Spp1* ^Low^ subclusters. F) Identification of enriched ligand‐receptor pairs between Cho‐*Spp1* ^High^ or Cho‐*Spp1* ^Low^ cholangiocyte subtypes with Mac‐C3‐*Trem2*. G) Dot plot demonstrating the expression levels of *Spp1‐Cd44* ligand‐receptor pair across distinct cell types. H) Representative images of multiplex immunofluorescence analysis of Spp1 (green) with Krt19 (red), Cd44 (green) with F4/80 (red), and DAPI (blue), in *Mettl3* ^ΔHep^ PSC compared with *Mettl3* ^fl/fl ^controls. Scale bars: 100 µm. I) Representative images of gross liver morphology, body weights, liver‐to‐body weight ratios, and serum biomarkers of *Spp1* ^ΔKrt19^ (*n* = 8) compared with *Spp1* ^fl/fl^ controls (*n* = 9) in the DDC‐induced PSC models. J) Representative images of multiplex immunofluorescence staining of Ecad (green) with Krt19 (red), Col1a1 (red), F4/80 (red), and DAPI (blue), in the DDC‐induced PSC of *Spp1* ^ΔKrt19^ compared with *Spp1* ^fl/fl^ controls. Data represent mean ± SEM; *p < 0.05, **p < 0.01, ***p < 0.001 by two‐tailed unpaired Student's t‐test. Scale bars: 100 µm.

CellChat analysis revealed enhanced interactions between Trem2^+^ macrophages and cholangiocytes in *Mettl3* ^△Hep^ PSC livers compared to *Mettl3* ^fl/fl^ liver tissues (Figure 5A; Figure S9A). To further explore which cholangiocyte subtypes interact with Trem2^+^ macrophages, t‐SNE plots identified two cholangiocyte subpopulations distinguished by *Spp1* expression levels (Figure 5B; Figure S9B). The Cho‐*Spp1* ^High^ subcluster exhibited greater developmental potential than the Cho‐*Spp1* ^Low^ subtype, as displayed by CytoTRACE analysis (Figure 5C). Notably, the Cho‐*Spp1* ^High^ subcluster also displayed higher expression of stemness‐related genes compared to the Cho‐*Spp1* ^Low^ subcluster (Figure S9B). Trajectory analysis revealed an enrichment of the stem‐like *Spp1* ^High^ cholangiocyte subpopulation at the origin of the trajectory (Figure 5D), accompanied by increased interactions between *Spp1* ^High^ cholangiocytes and Trem2^+^ macrophages in *Mettl3* ^△Hep^ PSC (Figure 5E). Ligand‐receptor analysis identified the Spp1‐Cd44 pair as the dominant interaction between *Spp1* ^High^ cholangiocytes and Trem2^+^ macrophages (Figure 5F). Dot plots and multiplex immunofluorescence co‐localization of Spp1 and Cd44 in *Mettl3* ^△Hep^ livers further validated this axis (Figure 5G,H; Figure S9C). Moreover, ELISA analysis indicated that Spp1 was highly secreted in the serum of the spontaneously and DDC‐induced *Mettl3* ^∆Hep^ PSC compared to *Mettl3* ^fl/fl^ control mice (Figure S9D). Cholangiocyte‐specific *Spp1* knockout (*Spp1* ^△Krt19^) alleviated DDC‐induced PSC progression, reducing ductular reactions and macrophage recruitment (Figure 5I,J; Figure S10A–C), underscoring the Spp1‐Cd44 axis as a critical pathogenic hub in PSC pathogenesis.

Previous studies have primarily focused on Spp1 expression in macrophages in the context of liver disease [23, 24].To elucidate the role of Spp1 in macrophages during PSC progression, we generated mice with myeloid cell deletion of Spp1 (*Spp1^△Lyz2^*) and subjected them to PSC and liver fibrosis model. In *Spp1^△Lyz2^* mice, disease progression was unaffected in the DDC‐induced PSC model (Figure S11A,B), whereas exacerbation was observed in the CCl_4_‐induced liver fibrosis. (Figure S11C,D). Collectively, these findings suggested that the Spp1‐Cd44 axis contributes to *Mettl3* ^△Hep^ driven PSC pathogenesis through cholangiocyte‐macrophage crosstalk.

### 2.6 Hepatic Mettl3 Deficiency Drives Macrophage Recruitment and Cholangiocyte Interaction via m6A‐Stabilized Mif and Csf1

**FIGURE 6:**
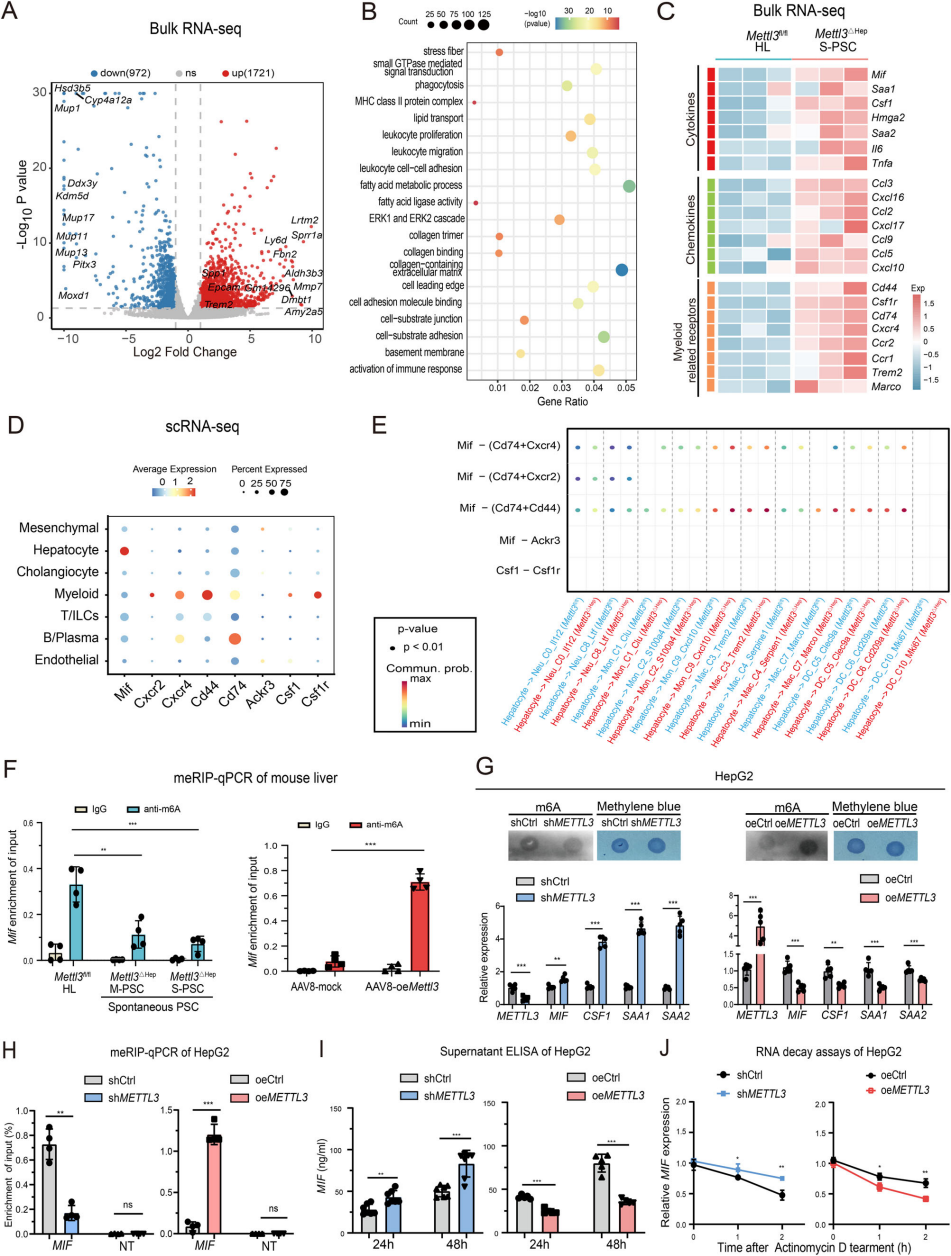
Hepatic *Mettl3* deficiency enhances Mif secretion, driving macrophage recruitment and cholangiocyte remodeling. A) Volcano plot depicting differentially expressed genes in the bulk RNA sequence analysis comparing *Mettl3* ^ΔHep^ S‐PSC with *Mettl3* ^fl/fl ^controls. The most significantly upregulated or downregulated genes were labeled on the volcano plot analysis. B) GO pathway enrichment analysis for *Mettl3* ^ΔHep^ S‐PSC versus *Mettl3* ^fl/fl ^controls. C) Heatmap demonstrating the expression levels of cytokines, chemokines, and myeloid related receptors in bulk RNA sequencing of *Mettl3* ^ΔHep^ S‐PSC compared to *Mettl3* ^fl/fl ^controls. D) Expression patterns of *Mif*, C*sf1*, and their corresponding receptors in the indicated cell subtypes from scRNA‐seq data *of Mettl3* ^ΔHep^ PSC. Color intensity indicates average gene expression, while dot size reflects the percentage of cells expressing the gene. E) Enrichment of *Mif* related ligand‐receptor pairs between hepatocytes and myeloid cell subtypes in *Mettl3* ^ΔHep^ S‐PSC compared to *Mettl3* ^fl/fl ^controls. F) m6A assays and RT‐qPCR of *METTL3, MIF, CSF1, SAA1, and SAA2* expression in HepG2 following *METTL3* knockdown (sh*METTL3*) or overexpression (oe*METTL3*), respectively. G) m6A‐RIP‐qPCR confirming Mettl3‐dependent post‐transcription regulation of *Mif* in spontaneous *Mettl3* ^ΔHep^ PSC compared with *Mettl3* ^fl/fl ^controls, as well as AAV8‐oe*Mettl3* versus AAV8‐mock in the DDC‐induced PSC model. H) m6A‐RIP‐qPCR analysis of *MIF* enrichment in HepG2 cells following *METTL3* knockdown (sh*METTL3*) or overexpression (oe*METTL3*). I, J) ELISA quantification of MIF concentration in the cell culture supernatants (I) and *MIF* RNA decay assays (J), conducted in HepG2 cells with *METTL3* knockdown (sh*METTL3*) or overexpression (oe*METTL3*), respectively. Data represent mean ± SEM; *p < 0.05, **p < 0.01, ***p < 0.001 by two‐tailed unpaired Student's t‐test.

Bulk RNA‐seq analysis of *Mettl3* ^△Hep^ S‐PSC and *Mettl3* ^fl/fl^ livers identified 1,721 upregulated and 972 downregulated genes (Figure 6A; Figure S12A). Pathway enrichment analysis (GO, GSEA, and KEGG) revealed activation of leukocyte proliferation and migration; cell‐cell adhesion, cell‐substrate junction and adhesion processes; lipid transport, and fatty acid metabolism (Figure 6B; Figure S12B). Consistent with our scRNA‐seq findings that demonstrated macrophage accumulation, bulk RNA‐seq of *Mettl3* ^△Hep^ PSC livers revealed significant upregulation of proinflammatory factors including cytokines (*Mif, Csf1, Saa1*, and *Saa2*) and chemokines (*Ccl3* and *Cxcl16*), as well as their corresponding receptors (*Cd44, Cd74*, and *Csf1r*), compared to *Mettl3* ^fl/fl^ counterparts (Figure 6C). These findings were further validated by qPCR assays (Figure S12C). Furthermore, single‐cell analysis revealed *Mif* expression predominantly enriched in hepatocytes, whereas its receptors (*Cd44, Cd74, Cxcr2, and Cxcr4)* were mainly expressed in myeloid cells. This expression pattern suggests the activation of a recruitment signaling pathway, potentially mediated through the *Mif‐(Cd74+Cd44)* axis (Figure 6D,E; Figure S12D,E). SRAMP analysis predicted multiple m6A modification sites within *Mif* and *Csf1* transcripts (Figure S13A). In vitro validation in HepG2 cells confirmed *METTL3* modulated the expression of MIF, CSF1, SAA1, and SAA2 by regulating global m6A levels; *METTL3* knockdown decreased m6A and upregulated these cytokines, whereas its overexpression had the opposite effects (Figure 6F).

As the catalytic core of the m6A methyltransferase complex, Mettl3 could directly mediate the m6A modification of mRNAs encoding the dysregulated cytokines observed in *Mettl3* ^△Hep^ livers. MeRIP‐qPCR assays demonstrated significantly reduced m6A enrichment on *Mif* and *Csf1* in *Mettl3* ^△Hep^ livers compared to *Mettl3* ^fl/fl^ controls, a phenomenon that was reversed by AAV8‐oe*Mettl3* in the DDC‐treated PSC model (Figure 6G; Figure S13B). In vitro studies using HepG2 cells confirmed METTL3‐dependent m6A dynamics of *MIf* and *CSf1* expression (Figure 6H; Figure S13C). Consistently, *METTL3* knockdown in HepG2 cells increased MIF secretion as measured by ELISA analysis, whereas *METTL3* overexpression significantly suppressed its expression (Figure 6I). Actinomycin D assays further revealed a prolonged *MIF* and CSF1 mRNA half‐life following *METTL3* knockdown and accelerated mRNA decay upon *METTL3* overexpression, establishing METTL3 as a regulator of MIF and CSF1 stability via m6A‐mediated RNA decay (Figure 6J; Figure S13D). In migration assays, conditioned medium from METTL3‐knochdown HepG2 enhanced THP‐1 cell recruitment. This effect was attenuated by neutralizing antibodies targeting MIF or CSF1, consistent with these cytokines mediating the enhanced migration (Figure S13E). In summary, METTL3 modulates the expression of hepatocyte‐derived MIF and CSF1 via m6A methylation, which promotes the recruitment of Trem2+ macrophages and facilitates cholangiocyte‐macrophage crosstalk, thereby contributing to PSC progression.

To evaluate the translational relevance of our findings from the mouse *Mettl3* ^△Hep^ PSC model, we interrogated existing scRNA‐seq and single nucleus RNA‐seq (snRNA‐seq) data from human PSC samples (GSE243981) [25]. Subcluster analysis identified distinct cell populations implicated in cholangiocyte‐macrophage crosstalk: an SPP1^+^ cholangiocyte subset (hCho_C2_SPP1) and a TREM2^+^ macrophage subset (hMac_C1_TREM2). The hMac_C1_TREM2 subset was significantly enriched in human PSC tissues (Figure S14A–H). Cell‐cell communication analysis further revealed a significantly enriched SPP1‐CD44 interaction between these subsets, corroborating our observations in *Mettl3* ^△Hep^ mice (Figure S15A–E). Consistent with this, spatial transcriptomic profiling of human PSC samples confirmed the coordinated upregulation of key genes within this pathway, including *SPP1, CD44, MIF, CSF1, CD74*, and *CSF1R* (Figure S15F).

### 2.7 Pharmacological Activation of Mettl3 Ameliorates PSC and Liver Fibrosis

**FIGURE 7:**
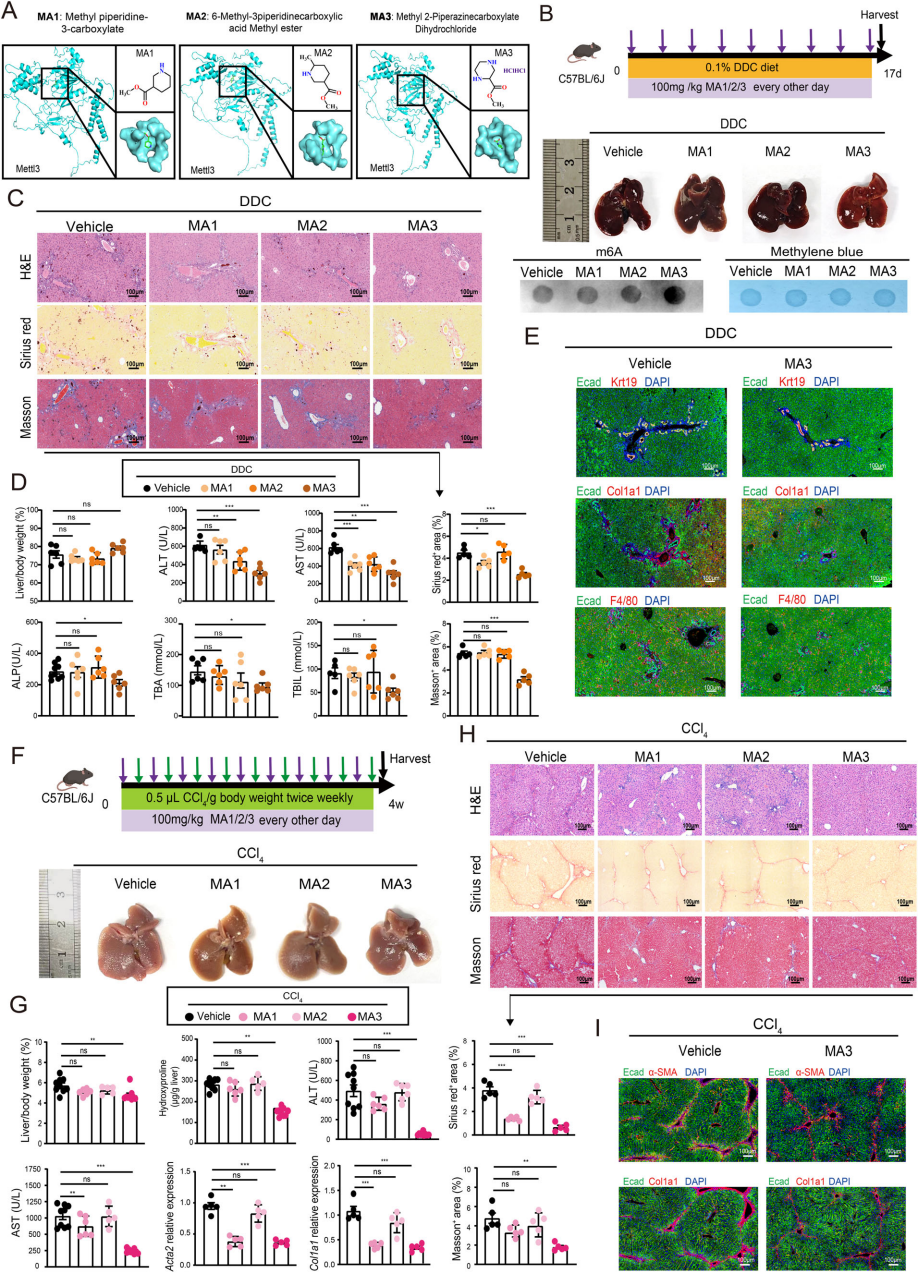
The Mettl3 activator MA3 significantly attenuated DDC‐induced PSC and liver fibrosis in vivo. A) Chemical structures of small molecular Mettl3 activators (MA1‐MA3) and the predicted interactions between Mettl3 and Mettl3 activators. B) Experimental design depicting the therapeutic efficacy of Mettl3 activators in the DDC‐induced PSC model, accompanied by representative images of gross liver morphology and m6A dot plots. C) Representative histopathology analyses using H&E, Sirius Red staining, and Masson's trichrome staining, with quantification of the positive area. D) Liver‐to‐body weight ratios and serum biomarkers of ALT, AST, ALP, TBA, and TBIL in the DDC‐induced PSC model following treatment with Mettl3 activators. E) Representative immunofluorescence staining displaying Ecad (green) alongside Krt19 (red), Col1a1 (red), F4/80 (red), with DAPI (blue); in the DDC‐induced PSC model treated with MA3. F) Experimental scheme of Mettl3 activators in the CCl_4_‐induced liver fibrosis model. G) Liver‐to‐body weight ratios, serum biomarkers of ALT and AST, and gene expression levels of *Acta2* and *Col1a1*, in the CCl_4_‐induced liver fibrosis model treated with Mettl3 activators. H) Representative histopathology analyses (H&E, Sirius Red, and Masson's staining) in the DDC‐induced PSC model after treatment with Mettl3 activators. The percentage of positive areas was quantified. I) Representative immunofluorescence staining showing Ecad (green) along with Col1a1 (red), F4/80 (red) in the CCl_4_‐induced fibrosis model treated with MA3. Data represent mean ± SEM; *p < 0.05, **p < 0.01, ***p < 0.001 by two‐tailed unpaired Student's t‐test.

While extensive research has focused on METTL3 upregulation in cancer and the development of its inhibitors, the exploration of METTL3 agonists remains limited [26], largely due to the absence of clear therapeutic contexts where METTL3 activation would offer a clinical advantage. The molecular docking analysis has identified three candidate Mettl3‐activating compounds (MA1, MA2, and MA3) (Figure 7A); however, their therapeutic efficacy against PSC remains uncharacterized [26]. In our DDC‐induced PSC models, MA3 treatment significantly increased total RNA m6A levels (Figure 7B) and mitigated ductular reaction (Krt19 and Krt7) (Figure S16A), ameliorated liver fibrosis (assessed via Sirius Red, Masson's trichrome staining, and decreased Hydroxyproline content) and serum injury markers (ALT, AST, ALP, and TBA), compared to control and the other two candidate compounds (Figure 7C–E; Figure S16B,C). Similarly, in CCl_4_‐induced liver fibrosis, MA3 also attenuated collagen deposition (hydroxyproline content), serum AST and ALT levels, and histopathological damage, with further validation reinforcing its anti‐fibrotic efficacy (Figure 7F–I; Figure S16D,E). Moreover, our results showed that MA3 treatment failed to ameliorate the pathological changes induced by DDC in the Mettl3^∆Hep^ mice, indicating that the therapeutic effect of MA3 is specifically dependent on Mettl3 expression in hepatocytes (Figure S17). These findings demonstrate that MA3 suppresses PSC and liver fibrosis by targeting ductular hyperplasia, extracellular matrix accumulation, and inflammatory responses across preclinical models.

**FIGURE 8:**
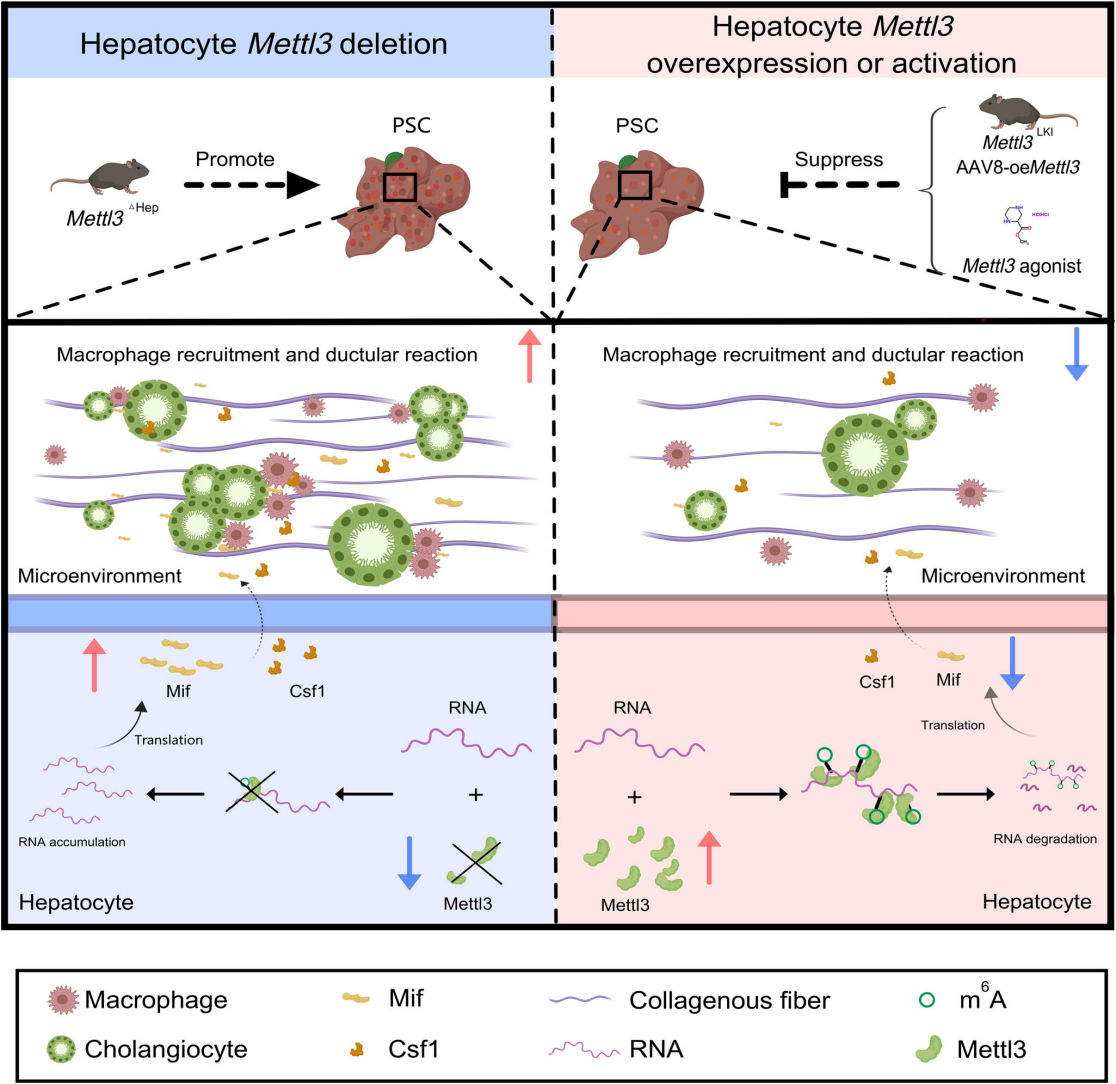
Mechanistic model: hepatocyte‐specific *Mettl3* deficiency promotes PSC progression through cholangiocyte‐macrophage interactions.

Collectively, our study establishes that hepatocyte *Mettl3* deficiency drives PSC progression by augmenting the m6A‐dependent secretion of Mif/Csf1, leading to Trem2^+^ macrophage infiltration, which in turn instigates pathogenic cholangiocyte‐macrophage interactions via the Spp1‐Cd44 pathway. Critically, restoring Mettl3 function, including *Mettl3* ^LKI^, AAV8‐mediated overexpression, or pharmacological activation, all effectively ameliorated ductular reactions, fibrotic remodeling, and inflammatory responses in DDC‐induced PSC models (Figure 8), highlighting a compelling therapeutic avenue for cholestatic liver diseases.

## 3 Discussion

Our study defines hepatocyte Mettl3 as a pivotal regulator of cholangiopathy and unveils epitranscriptomic remodeling as a therapeutic vulnerability in PSC pathogenesis. In contrast to existing therapies that have failed to halt PSC progression [27], our multimodal approach— employing viral delivery, genetic knock‐in, or pharmacological activation of Mettl3—effectively alleviated biliary injury and fibrosis. These findings position m6A modulation as a promising therapeutic strategy for PSC treatment.

The role of Mettl3 in liver development and injury remains controversial according to recent studies. Li et al. reported that hepatocyte‐specific *Mettl3* CKO mice displayed no apparent abnormalities under normal diet conditions but exhibited aggravated NASH progression upon high‐fat diet (HFD) challenge [18]. In contrast, Xu et al. and Wang et al. observed pronounced phenotypes in hepatocyte‐specific *Mettl3* CKO mice under normal diet conditions, including hepatocyte apoptosis, ballooning, and steatosis, leading to liver injury and postnatal lethality [20, 21]. Notably, Xu et al. further demonstrated that depleting *Mettl3* in adult hepatocytes using Alb‐CreERT2 did not disrupt liver homeostasis [21], whereas Wang et al. found that administering AAV8‐ Alb‐Cre virus to 1‐week‐old *Mettl3* ^fl/fl^ mice had no discernible impact on postnatal liver development [20]. However, Yao et al. reported that adult hepatocyte‐specific *Mettl3* knockout, using *Mettl3* ^fl/fl^ mice injected with AAV8‐TBG‐Cre, exacerbated alcohol‐associated steatohepatitis (ASH), enhancing pathological features including steatosis and neutrophil infiltration [28].Our data show that embryonic *Mettl3* ablation in hepatocytes (*Mettl3* ^ΔHep^) caused spontaneous and heterogeneous PSC, but its deletion in adult hepatocytes failed to promote the disease. Notably, restoring or activating Mettl3 in adult hepatocytes effectively mitigated DDC‐induced cholestatic injury and liver fibrosis. The contrasting outcomes between embryonic and adult *Mettl3* deletion suggest a differential therapeutic threshold for Mettl3 activity during embryonic liver development versus adult liver cholestatic fibrosis.

During embryogenesis, Mettl3 is highly expressed in hepatic progenitor cells and embryonic hepatocytes, whereas Mettl3‐mediated m6A methylation is essential for hepatic lineage differentiation and organogenesis, relying on tightly regulated epitranscriptomic control [20]. In contrast, adult hepatocytes—which exhibit lower steady‐state Mettl3 expression—tolerate partial Mettl3 loss under both homeostatic and injury conditions. Although basal *Mettl3* levels in adult hepatocytes are sufficient for routine function, counteracting PSC progression appears to require supra‐physiological Mettl3 activation. This threshold model aligns with growing evidence that m6A methylation dynamically regulates stress‐responsive pathways in liver fibrosis, including TGF‐β signaling, epithelial‐mesenchymal transition, inflammatory cytokine production, and HSC activation [29, 30]. By amplifying Mettl3 activity beyond baseline, our interventions may shift the epitranscriptomic equilibrium toward resolution of fibrotic programs. Notably, all three therapeutic strategies —genetic knock‐in, AAV8‐mediated delivery, and pharmacological Mettl3 activation—converged to ameliorate PSC, highlighting the potential of Mettl3 agonist as a robust antifibrotic approach.

Challenging the prevailing cholangiocyte‐centric view of cholestatic injury [31, 32, 33], our findings delineate a novel hepatocyte‐initiated triad pathway in which hepatocyte‐specific* Mettl3* deficiency alters m6A‐dependent RNA metabolism, leading to the upregulation of inflammatory mediators such as Mif and Csf1. This cascade promotes the recruitment of Trem2^+^ macrophages, which interact with Spp1^high^ cholangiocytes via the Cd44‐Spp1 axis, thereby sustaining a pro‐fibrotic feedback loop [34]. These results expand the functional scope of m6A modification beyond its established roles in metabolic disorders such as NASH and liver injury, where hepatocyte specific m6A dysregulation exacerbates insulin resistance and lipid accumulation [12]. Previous studies have emphasized macrophage‐derived Spp1 in metabolic dysfunction‐associated steatohepatitis, liver fibrosis, and PSC [24, 35, 36, 37], where it is largely restricted to lipid‐associated macrophages (LAMs) [38], and drives fibrotic remodeling by activating hepatic stellate cells [39, 40]. In contrast, we demonstrate that in both spontaneous or DDC‐induced cholestatic models *of Mettl3* ^ΔHep^ mice, Spp1 is predominantly derived from cholangiocytes and amplifies peribiliary inflammation by engaging Trem2^+^ macrophages, thereby driving PSC progression. Although certain macrophage subsets do exhibit elevated Spp1 expression relative to other macrophage subpopulations in liver diseases, their contribution remains negligible at single‐cell resolution compared to the robust Spp1 production observed in cholangiocytes. In liver pathologies lacking a prominent biliary reaction, myeloid‐derived Spp1 may exert protective effects in nonalcoholic steatohepatitis [35] and fibrosis. However, under conditions marked by significant cholangiocyte proliferation or overt biliary reactions such as PSC, cholangiocyte‐derived Spp1 emerges as a pivotal driver of disease progression. Furthermore, CellChat analysis revealed that the Spp1‐Cd44 axis mediates crosstalk between Spp^high^ cholangiocytes and Trem2^+^ macrophages, although it should be noted that not all Cd44 isoforms bind Spp1.

Current research on METTL3 predominantly focuses on its upregulated expression in human cancers and the subsequent development of METTL3 inhibitors, such as STM2457, to impede tumorigenesis and cancer progression [41, 42, 43]. In contrast, the therapeutic potential of Mettl3 activators remains largely unexplored, partly due to the absence of established pharmacological contexts for their application. Our findings demonstrate that hepatocyte‐specific *Mettl3* knockout spontaneously induces PSC‐like pathology, whereas hepatic specific *Mettl3* knock‐in, AAV8 mediates Mettl3 overexpression, or pharmacological activation all effectively attenuate PSC and liver fibrosis in experimental models. These findings underscore the significant therapeutic potential of Mettl3 activators for non‐oncological liver diseases. Notably, the small‐molecule Mettl3 agonist (MA3) showed efficacy in preclinical models of DDC‐induced PSC and CCl_4_‐induced liver fibrosis, without evidence of hepatotoxicity. Structural optimization of MA3 could further improve its potency and safety. Future investigations should explore potential synergies between MA3 and existing PSC therapies, particularly ursodeoxycholic acid (UDCA), in disrupting the Spp1‐Cd44 signaling axis, and evaluate whether hepatocyte m6A signatures could serve as dynamic biomarkers for cholestatic liver disease [7]. Nonetheless, several limitations warrant consideration. Although murine preclinical models emphasize Mettl3 activation and restoration as promising strategies, DDC‐induced cholestasis in mice dose not fully recapitulate the immunopathology of human PSC [25, 44]. Clinical validation will be essential to assess the safety and efficacy of METTL3 activation in humans, especially given that chronic METTL3 activation requires careful evaluation in light of its association with hepatocellular carcinoma [12, 45]. Additionally, it remains unclear whether Mettl3 in hepatocytes exerts m6A‐independent functions that may contribute to PSC pathogenesis. Further investigation is also needed to elucidate the interplay between Mettl3 and other m6A regulators, such as Fto and Alkbh5, whose dysregulation has been implicated in ferroptosis‐associated liver injury and HFD‐induced steatotic liver disease [46, 47, 48].

In conclusion, this study establishes hepatocytes as pivotal regulators of PSC pathogenesis and highlights hepatocyte Mettl3 as a promising therapeutic target. Pharmacological activation of Mettl3 represents a potential strategy for developing targeted therapies that can reverse PSC progression.

## 4 Experimental Section

### 4.1 Ethics Statement

All procedures were approved by the Science and Technology Ethics Committee of Beijing University of Technology.

### 4.2 Animals

All mice were maintained on a C57BL/6J background and housed under specific pathogen‐free (SPF) conditions, with a 12‐h light/dark cycle, ambient temperature of 24°C ±2°C, relative humidity of 30%–70%, and ad libitum access to food and water. Hepatocyte‐specific *Mettl3* knockout (*Mettl3* ^△Hep^) mice were generated by crossing *Mettl3* ^fl/fl^ mice with Alb‐Cre mice. Hepatocyte‐specific *Mettl3* Knock‐in (*Mettl3* ^LKI^) mice were generated by crossing Rosa26‐lsl‐*Mettl3* mice with Alb‐CreERT2 mice. Cholangiocyte‐specific inducible knockout of *Spp1* mice (*Spp1* ^△Krt19^) were generated by crossing *Spp1* ^fl/fl^ mice with Krt19‐CreERT2 mice. *Mettl3* ^LKI^ and *Spp1* ^△Krt19^ mice were treated with tamoxifen (Sigma‐Aldrich, T5648) at 1 mg/mouse for 5 consecutive days via intraperitoneal injection, while control mice received an equivalent volume of corn oil (BioPremium, ST1177). Mouse suppliers and primers used for genotyping are listed in Table S1.

### 4.3 DDC‐Induced Mouse PSC Model

Mice were fed a diet containing 0.1% 3,5‐diethoxycarbonyl‐1,4‐dihydrocollidine (DDC, Trophic Animal Feed High‐tech, China) for 17 days to induce primary sclerosing cholangitis. On day 17, the mice were euthanized and body and liver weights were recorded to evaluate the phenotype. Serum and liver tissues were collected for the analysis of cholestasis, fibrosis, and histopathology [44].

### 4.4 CCl_4_‐Induced Hepatic Fibrosis Model

Mice administered intraperitoneal injections of 15% CCl_4_ in corn oil (0.5 µL/g body weight) twice weekly for 4 weeks. Control mice received intraperitoneal injections of an equivalent volume of corn oil alone. Body and liver weights were recorded, and serum as well as liver tissues were harvested for fibrosis and histopathological assessment [49].

### 4.5 AAV8 Virus Preparation and Transduction

Serotype 8 adeno‐associated virus (AAV8) vectors were generated by cloning the *Mettl3* coding sequence downstream of thyroxine‐binding globulin (TBG) promoter to achieve hepatocyte specific expression. Viral particles were packaged by Weizhen Biosciences Inc. (Jinan, China) with a titer >2.0 ×10^13^ viral genomes/mL. For in vivo AAV8 mediated Mettl3 transduction, 1.0×10^11^ viral genomes per mouse of either AAV8‐control (AAV8‐mock) or AAV8‐oe*Mettl3* were administered via tail vein injection into 5‐week‐old C57BL/6J mice for further histopathology analysis.

### 4.6 Hydroxyproline Assay

Mouse liver tissue fragments were weighed and homogenized in ultrapure water (100 µL/sample), hydrolyzed in 30% HCl at 100°C overnight, centrifuged (10 000 g, 3 min), and filtered through a 40 µm membrane. Supernatants (25 µL) were transferred to a 96‐well untreated flat‐bottom microplate, and dried for 75 min at 60°C. Hydroxyproline content was quantified using a Hydroxyproline Assay Kit (Servicebio, G4311‐48T), according to the manufacturer's guidelines.

### 4.7 RNA Isolation and qRT‐PCR Assays

Total RNAs from mouse liver tissues were extracted using TRIzol reagent (Life Technologies Corporation, CA). Complementary DNA (cDNA) was synthesized using HiScript III RT SuperMix for qPCR (Vazyme, China). Quantitative reverse transcription‐polymerase chain reaction (qRT‐PCR) was performed utilizing ChamQ Universal SYBR qPCR Master Mix (Vazyme) on a LightCycler 480 Real‐Time PCR System (Roche, Switzerland). The Cycling conditions were as follows: pre‐denaturation at 95°C for 30 s, denaturation at 95°C for 10 s, annealing and extension at 60°C for 30 s; with 40 cycles in total. The relative gene expression levels were normalized to Gapdh via the 2–^ΔΔCT^ method. Primer sequences are listed in Table S2.

### 4.8 m6A Dot Blot Assay

Total RNAs were isolated as described in the preceding section. Subsequently, RNA samples were dissolved in three volumes of RNA incubation buffer. Aliquots (500 ng) were denatured at 65°C for 5 min and then blotted onto an Amersham Hybond‐N+ membrane (GE Healthcare, USA) using a Bio‐Dot Apparatus (Bio‐Rad, USA), with ice‐cold 20× SSC buffer (Sigma‐Aldrich, Germany). Membranes were UV‐crosslinked for 5 min, washed with PBST (0.1% Tween‐20), and stained with 0.02% Methylene blue (Sangon Biotech, China) to verify RNA loading. After blocking with 5% non‐fat milk for 1 h at room temperature (RT), membranes were incubated overnight at 4°C with a specific m6A antibody (1:1000; Millipore, Abcam, ab151230). Following PBST washes, blots were incubated with HRP‐conjugated anti‐mouse IgG (1:5000) for 1 h at RT and visualized using an imaging system (Bio‐Rad, USA).

### 4.9 Western Blot Analysis

Proteins from mouse liver tissues were extracted using RIPA lysis buffer (Beyotime, China) supplemented with protease and phosphatase inhibitor cocktails. Western blot analysis was performed following established protocols using antibodies against α‐SMA, Col1a1, Pdgfrβ, and Gapdh. Briefly, protein samples were mixed with SDS sample buffer and boiled for 10 min to denature the protein. Subsequently, equal amounts of protein were then loaded into each well of the gel. Electrophoresis was carried out at 100 V for 60 min. The separated proteins were subsequently transferred from the gel to a PVDF membrane. After blocking for 1 h at room temperature, the membranes were incubated with the primary antibody and secondary antibodies, respectively. Finally, proteins were detected using a chemiluminescence western blot substrate (Tanon 5200, China). Image analysis was conducted using ImageJ software (NIH, USA). Antibody details were provided in Table S3.

### 4.10 RNA Decay Assays

HepG2 cells were seeded in 6‐well plates at a density of 2×10^5^ cells per well and treated with Actinomycin D (5 µg/mL; A9415, Sigma–Aldrich, USA) for the specific durations. Total RNAs were extracted using TRIzol reagent (Life Technologies, USA) and subsequently analyzed by RT‐qPCR.

### 4.11 Sirius Red Staining

Neutral‐buffered formalin‐fixed, paraffin‐embedded tissue sections were dewaxed in xylene and rehydrated through a graded ethanol series. Nuclei were counterstained with celestine blue solution for 5–10 min, rinsed in distilled water, and then incubated with Picro‐Sirius Red solution (0.1% Sirius Red in saturated picric acid; 15–30 min). Subsequently, the sections were dehydrated in absolute ethanol (three changes), cleared in xylene (two changes), and mounted with neutral resins.

### 4.12 Masson's Trichrome Staining

Rehydrated tissue sections were mordanted in Bouin's solution overnight at room temperature and then thoroughly rinsed under running water for 10 min. The sections were stained with Weigert's iron hematoxylin for 5 min, differentiated in 1% acid alcohol for 30 s, and blued in saturated lithium carbonate solution for 30 s. After rinsing, the sections were stained with Biebrich scarlet‐acid fuchsin for 5–10 min, treated with 1% phosphomolybdic acid solution for 5 min, and directly counterstained with aniline blue for 5 min without washing. Subsequently, the sections were then immersed in 1% glacial acetic acid for 1 min, dehydrated through three changes of 95% ethanol followed by absolute ethanol, cleared in xylene, and finally mounted with synthetic resin.

### 4.13 Immunohistochemical (IHC) Staining

Paraffin sections underwent antigen retrieval via microwave irradiation in citrate buffer (pH 6.0), followed by blocking endogenous peroxidase with 3% H_2_O_2_ for 15 min and non‐specific blocking with 10% goat serum for 1 h at RT. Sections were then incubated overnight at 4°C with the following primary antibodies: α‐SMA, Col1a1, Krt19, Ki67, F4/80, Krt7, Cd31, Cd163, and Marco. After rinsing with PBS, sections were incubated with biotinylated secondary antibodies (1:200; ZSBIO, PV‐6001) for 1 h at RT, followed by diaminobenzidine (DAB, ZSGB‐BIO, ZLI‐9019), and the reactions were terminated by rinsing with distilled water. Nuclei were counterstained with hematoxylin (Vector Laboratories, H‐3404) for 5 min, dehydrated through graded ethanol, cleared in xylene, and mounted with neutral resin.

### 4.14 Multiplex Immunofluorescent Labeling

To investigate the co‐expression and spatial distribution of Spp1 and Cd44; Krt19 and F4/80; Cd44 and F4/80; and Krt19 and Mettl3 in mouse liver tissues, sequential multiplex immunofluorescence was performed using the PANO 3‐plex immunohistochemistry kit (0004100100, Panovue). Mouse liver tissue sections underwent antigen retrieval according to the kit instructions. Primary antibodies target the respective antigens were applied sequentially in the following detection cycles: sections were incubated with the appropriate primary antibody cocktail, followed by horseradish peroxidase (HRP)‐conjugated secondary antibodies. Target signals were then amplified using tyramide signal amplification (TSA) with fluorophore‐conjugated tyramides. Antibody stripping was conducted after each TSA reaction by microwave‐mediated heat treatment (specific conditions) to remove bound antibodies while preserving the deposited fluorophores. Upon completion of all antigen labeling cycles, nuclei were counterstained with 4′,6‐diamidino‐2‐phenylindole (DAPI) for 15–20 min. Finally, sections were mounted using Fluoroshield Mounting Medium (Cat# ZLI‐9556, ZSBIO) and imaged with the TissueFAXS Plus imaging system (TissueGnostics, Austria). Antibodies used in this study are listed in Table S3.

### 4.15 Bulk RNA‐seq Analysis

RNA was extracted from liver tissues of Mettl3^fl/fl^ HL (*n* = 3), Mettl3^△Hep^ S‐PSC (*n* = 3) using TRIzol reagent (Life Technologies). The cDNA library was prepared by the Beijing Genomics Institute (Beijing, China). The paired‐end reads were generated by the Illumina HiSeq 2500 platform supplied by the Beijing Genomics Institute. Clean reads were aligned to the GRCm38 (mm10) reference mouse genome using HISAT2. Transcript abundance (expected counts or TPM/FPKM) was quantified using RSEM (v1.2.8) based on the alignments. Differentially expressed genes (DEGs) between the *Mettl3* ^△Hep^ S‐PSC and *Mettl3* ^fl/fl^ groups were identified using RSEM output within a differential expression analysis framework. DEGs were identified using the “edgeR” R package, applying a significance threshold of false discovery rate (FDR) ≤ 0.05 and absolute value of log2 fold change (FC) ≥ 1.

### 4.16 Methylated RNA Immunoprecipitation Followed by qPCR (MeRIP‐qPCR)

Total RNA was extracted from liver tissues of conditional knockout (CKO), and corresponding control mice using TRIzol reagent (Thermo Fisher Scientific), following the manufacturer's protocol for the Magna MeRIP m6A Kit (Millipore, USA).100 µg of total RNA per sample was fragmented and incubated overnight at 4°C with magnetic beads conjugated to anti‐m⁶A antibody or control IgG antibody with continuous rotation. Following immunoprecipitation, RNA was purified according to the kit instructions. For relative quantification analysis, the ∆CT value was calculated using the CT value of the target group, with the CT value of the IgG group as the reference. Relative quantification was determined by 2^‐∆CT^. Data are presented as mean relative enrichment ± SEM of biological replicates, each assayed in three technical replicates.

### 4.17 Cell Culture and Transfection

The HepG2 cells were cultured in DMEM medium (Gibco, USA) supplemented with 10% fetal bovine serum (FBS, Gibco) in an incubator at 37°C with 5% CO_2_. Short hairpin RNAs (shRNAs) targeting human *METTL3* were designed using the online RNAi Vector designer tool (VectorBuilder). A non‐targeting scrambled shRNA (shCtrl) served as the negative control. The shRNA sequences(GCTGCACTTCAGACGAATTAT)of human *METTL3* were cloned into the pLKO.1‐GFP plasmid backbone. The full‐length human *METTL3* cDNA (NM_019852.5)was cloned into the pLV‐GFP plasmid for overexpression. Lentiviral particles were produced by co‐transfecting HEK293T cells with the *METTL3* shRNA or *METTL3* overexpression plasmid, and the packaging plasmids psPAX2 and pMD2.G using Lipofectamine 2000 (Thermo Fisher Scientific) according to the manufacturer's protocol. Viral supernatants were collected 48 h post‐transfection, filtered through a 0.45 µm filter, and concentrated with PEG8000. HepG2 cells were transduced with lentivirus in the presence of 8 µg/mL Polybrene. Stable cell lines were constructed using FACS sorting of GFP positive cells. Knockdown or overexpression efficiency of *METTL3* was confirmed by qRT‐PCR and Western blotting.

### 4.18 Enzyme‐Linked Immunosorbent Assay (ELISA)

HepG2 cell culture supernatants were collected at 24 or 48 h post‐transfection. The supernatants were centrifuged (300 × g for 5 min) to remove cellular debris and stored at −80°C until analysis. Macrophage migration inhibitory factor (Mif) concentration was quantified in the supernatants using a commercial ELISA kit (Beyotime, PM715) according to the manufacturer's protocols. Absorbance readings were measured using a microplate reader SpectraMax iD5. All samples were assayed in triplicate.

### 4.19 Single‐Cell RNA‐seq

To minimize technical batch effects, single‐cell suspensions from three mice within the same experimental group were pooled in equal proportions to form one combined sample prior to single‐cell capture. Liver tissues were collected from *Mettl3* ^fl/fl^ mice (*n* = 2, liver tissues from three mice were pooled as one test) and *Mettl3* ^ΔHep^ S‐PSC(*n* = 1, liver tissues from three mice were pooled as one test). Immediately following surgical resection, tissues were placed on ice and rinsed with ice‐cold phosphate‐buffered saline (PBS). The tissues were minced into ∼1 mm fragments and digested using mouse Liver Dissociation Kit (Miltenyi Biotec,130‐105‐807) at 37°C for 30 min with gentle agitation. Digestion was halted by adding PBS containing 2% fetal bovine serum (FBS). The cell suspension was sequentially filtered through 40 µm strainers, centrifuged (e.g., 300 × g, 5 min, 4°C), and resuspended in PBS with 2% FBS. Erythrocytes were lysed using ACK Lysing Buffer for 5 min. Cells were washed twice and resuspended in PBS containing 0.1% BSA. Viable cell concentration and viability (>80%) were assessed by Trypan Blue exclusion using a Countess II FL Automated Cell Counter (Thermo Fisher Scientific), ensuring sample quality met the recommendations of 10x Genomics. Single‐cell RNA libraries were constructed using the 10× Genomics Chromium Controller platform with the Chromium Single Cell 3′ library kit in Capitalbio Technology Corporation (China). Following the standard protocol, the constructed cDNA libraries were sequenced on the Illumina Nova Seq 6000 system. During the sequencing process, cell‐specific gene expression data were obtained by identifying the barcodes and unique molecular identifiers (UMIs) associated with each cDNA fragment [50].

### 4.20 Single‐Cell Transcriptomic Analysis

The raw sequencing data were initially processed using the Cell Ranger software (10× Genomics). The FASTQ files were aligned to the GRCh38 reference genome to generate the single‐cell gene expression matrix. Subsequently, the data were further processed and analyzed using the “Seurat” R package (version 4.3.0.1). Initially, a stringent quality control procedure was applied: cells with the number of expressed genes in the lowest 2% or highest 2% of the distribution, or with mitochondrial gene content exceeding 20%, were excluded. Next, the “DoubletFinder” R package was employed to remove potential doublets. Following quality control, the “NormalizeData” function was used to normalize the matrix, followed by “ScaleData” for data scaling. To reduce data dimensionality, principal component analysis (PCA) was employed, and batch effects across different samples were corrected using the “harmony” R package. For cell clustering, a graph‐based clustering algorithm was applied using the “FindNeighbors” and “FindClusters” functions, setting the resolution parameter to 1.2. Finally, t‐Distributed Stochastic Neighbor Embedding (t‐SNE) was employed to further reduce dimensionality and visualize the cell clusters in a two‐dimensional space [51].

### 4.21 Differential Gene Expression Analysis and Cell Annotation

To identify the DEGs across all clusters, the “FindAllMarkers” function from the “Seurat” R package was employed using default parameters (only.pos = TRUE, min.pct = 0.25), with statistical significance defined by the Wilcoxon rank‐sum test. Cell types were subsequently annotated based on the identified DEGs and the expression levels of established markers, including Endothelial cells (*Pecam1, Kdr*, and *Cdh5*), B/Plasma cells (*Cd79a, Mzb1*, and *Ms4a1*), T /Innate lymphoid cells (ILCs) (*Cd3e*, *Cd3d*, and *Nkg7*), Myeloid cells (*Aif1, Ccl6*, and *Cd80*), Cholangiocytes (*Tm4sf4*, *Spp1*, and *Epcam*), Hepatocytes (*Apoc3, Apoc1*, and *Fabp1*), Mesenchymal cells (*Col1a1*, *Rgs5*, and *Pdgfra*). Finally, the “DoHeatmap”, “VlnPlot”, and “DotPlot” functions from the “Seurat” R package were used to visualize marker expression patterns.

### 4.22 Functional Enrichment Analysis

Functional enrichment analysis was performed to identify key pathways between the control and *Mettl3* ^ΔHep^ S‐PSC groups. Gene Set Enrichment Analysis (GSEA) was conducted using the “mh.all.v2024.1.Mm.symbols.gmt” and “m5.all.v2024.1.Mm.symbols” gene lists obtained from the Molecular Signatures Database (MSigDB) database (https://www.gsea‐msigdb.org/gsea/msigdb/index.jsp) [52]. For the DEGs, Kyoto Encyclopedia of Genes and Genomes (KEGG) pathway and Gene Ontology (GO) enrichment analyses were performed via the “clusterProfiler” R package to identify significant enriched pathways. The terms with a p‐value < 0.05 were considered statistically significant. Finally, the “ggplot2” and “enrichplot” R packages were utilized to visualize the results.

### 4.23 Cell‐Cell Communication Analysis

To comprehensively elucidate the interaction networks among cell subtypes, the “CellChat” R package (version 1.6.1) was employed to quantify the cell‐cell communication networks [53, 54]. The “CellChatDB.mouse” database was utilized as the reference date set for ligand‐receptor interactions. Following the standardized workflow, the communication patterns between different cell types under various experimental conditions were systematically investigated [55].

### 4.24 Pseudotime Analysis

To comprehensively evaluate the differentiation dynamics of cell subtypes, the R packages “CytoTRACE” and “Monocle” were employed [56]. Initially, the developmental potential of each subpopulation was quantified with CytoTRACE. Subsequently, the Monocle method was utilized to reconstruct the cell differentiation trajectories. Specifically, the “differentialGeneTest” method was used to identify DEGs from each cluster, and genes with a *q*‐value < 0.00001 were selected to order the cells in the pseudotime analysis. Additionally, the trajectories were constructed using the “DDRTree” algorithm.

### 4.25 Statistical Analysis

All analyses were performed using Prism software or the R environment (version 4.4.1). Two‐tailed tests were applied for all statistical comparisons. Differential expressed genes between the two groups were identified using the Wilcoxon rank sum test in the ScRNA‐seq analysis. All data are presented as means± SEM; ^*^ *p* < 0.05, ^**^ *p* < 0.01, ^***^ *p* < 0.001 by two‐tailed unpaired Student's t‐test.

## Funding

The work was supported by National Natural Science Foundation of China (82173183, 82203636, 82470115, and 82271628), R&D program of Beijing Municipal Education Commission (KZ202210005010), The Fundamental Research Funds for Beijing Municipal Universities (05500054625503).

## Conflicts of Interest

The authors declare no conflict of interest.

## Author Contributions

W.T.P., Y.T.Y., Y.S.L., G.N.S., and M.Z. performed investigation, acquisition of data, validation, formal analysis, writing – original draft. L.F.W., Z.Y., W.L.Z, L.Y.L., Q.Z.Q., X.P.C, Y.L.N, H.X.C., and X.M.G. performed investigation, Methodology, and acquisition of data. W.Z.S provided clinical samples and helped with the clinical consultation. S.C.L., J.C., Y.L.W., B.L., and X.L.Y. performed study concept, funding acquisition, validation, writing, review and editing. All authors have approved the article.

## Supporting information




**Supporting File**: advs73418‐sup‐0001‐SuppMat.docx.

## Data Availability

The raw sequencing data of scRNA‐seq and bulk RNA sequencing have been deposited in the Genome Sequence Archive at the National Genomics Data Center (Beijing, China): PRJCA041622.
