# Supplementary material for: Hepatocyte Mettl3 Deficiency Drives Primary Sclerosing Cholangitis and Liver Fibrosis via Cholangiocyte‐Macrophage Crosstalk
Source: Adv Sci (Weinh). 2025 Dec 22;13(13):e12799. doi: 10.1002/advs.202512799 (PMC12955918; doi:10.1002/advs.202512799)
Supplement: Supplementary file 1 — Supporting File: advs73418‐sup‐0001‐SuppMat.docx. [file ADVS-13-e12799-s001.docx]

**Supporting Information**

**Hepatocyte Mettl3 Deficiency Drives Primary Sclerosing Cholangitis and Liver Fibrosis via Cholangiocyte-Macrophage Crosstalk**

Wenting Pan^1,2^ ^‡^, Yuting Yong^1,2‡^, Yuanshuai Li^1,2 ‡^, Gaona Shi^3‡^, Min Zhang^4‡^, Lingfei Wan^1,2^, Yue Zhao^1,2^, Wenling Zhan^1,2^, Yanli Lin^5^, Qiaozhen Qin^1^, Xupeng Chen^1,2^, Yanli Ni^2^, Haixu Chen^6^, Wenzai Shi^7^, Xiaomeng Guo^1^, Juan Chen^8^*, Shuchen Liu^9^*, Youliang Wang ^5^*, Bing Liu^2^*, Xinlong Yan^1^*

**This file includes:**

Supplementary materials and methods

Supplementary Figures. S1 to S17

Supplementary Tables. S1 to S3

**Supporting Information Figures and Figure Legends**


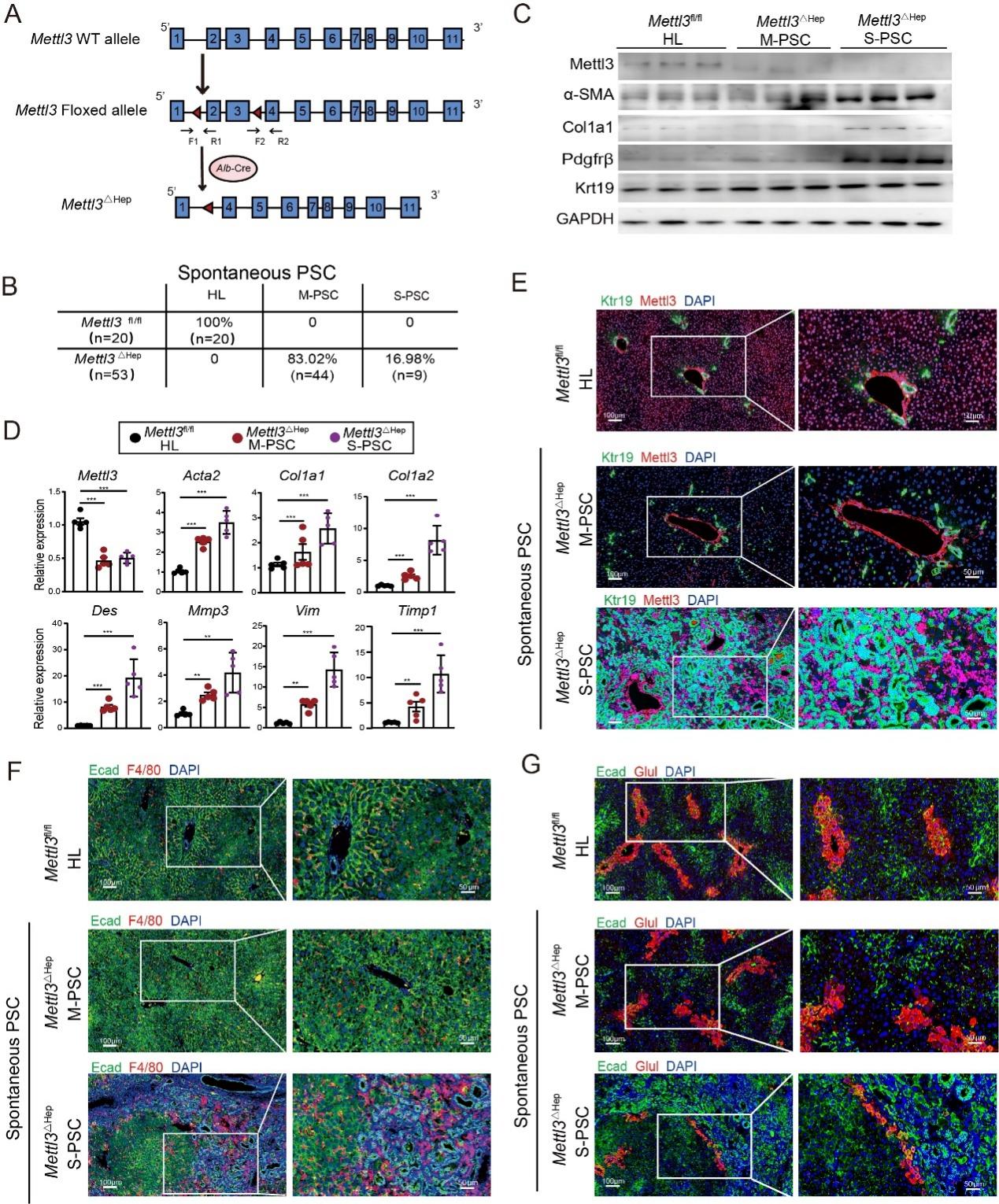


Figure S1. Generation and phenotypic characterization of hepatocyte- specific *Mettl3* knockout mice.

A) Schematic illustrating the targeting strategy for generating hepatocyte-specific *Mettl3* knockout (*Mettl3*^△Hep^) mice. B) Incidence of spontaneous mild PSC (*Mettl3*^△Hep^ M-PSC, *n*=44) and severe PSC (*Mettl3*^△Hep^ S-PSC, *n*=9) in *Mettl3*^△Hep^ mice (*n*=53). C) Protein levels of Mettl3, α-SMA, Col1a1, Pdgfrβ, and Krt19 in *Mettl3*^fl/fl^ HL, *Mettl3*^△Hep^ M-PSC, and *Mettl3*^△Hep^ S-PSC liver tissues. D) RT-qPCR validation of *Mettl3* expression and fibrosis-related marker genes (*Acta2, Col1a1, Col1a2, Des, Mmp3, Vim,* and *Timp1*) in indicated groups. E) Representative images of immunofluorescence analysis of Krt19 (green) and Mettl3 (red) in liver sections. F, G) Representative images of immunofluorescence analysis of Ecad (green) and F4/80 (red) (F); Ecad (green) and Glul (red) (G), in *Mettl3*^fl/fl^ HL, *Mettl3*^△Hep^ M-PSC, and *Mettl3*^△Hep^ S-PSC. Scale bars: 100 μm. Data represent mean ± SEM; *P < 0.05, **P < 0.01, ***P < 0.001 by two-tailed unpaired Student’s t-test. M-PSC: mild-primary sclerosing cholangitis; S-PSC: severe-primary sclerosing cholangitis.

**
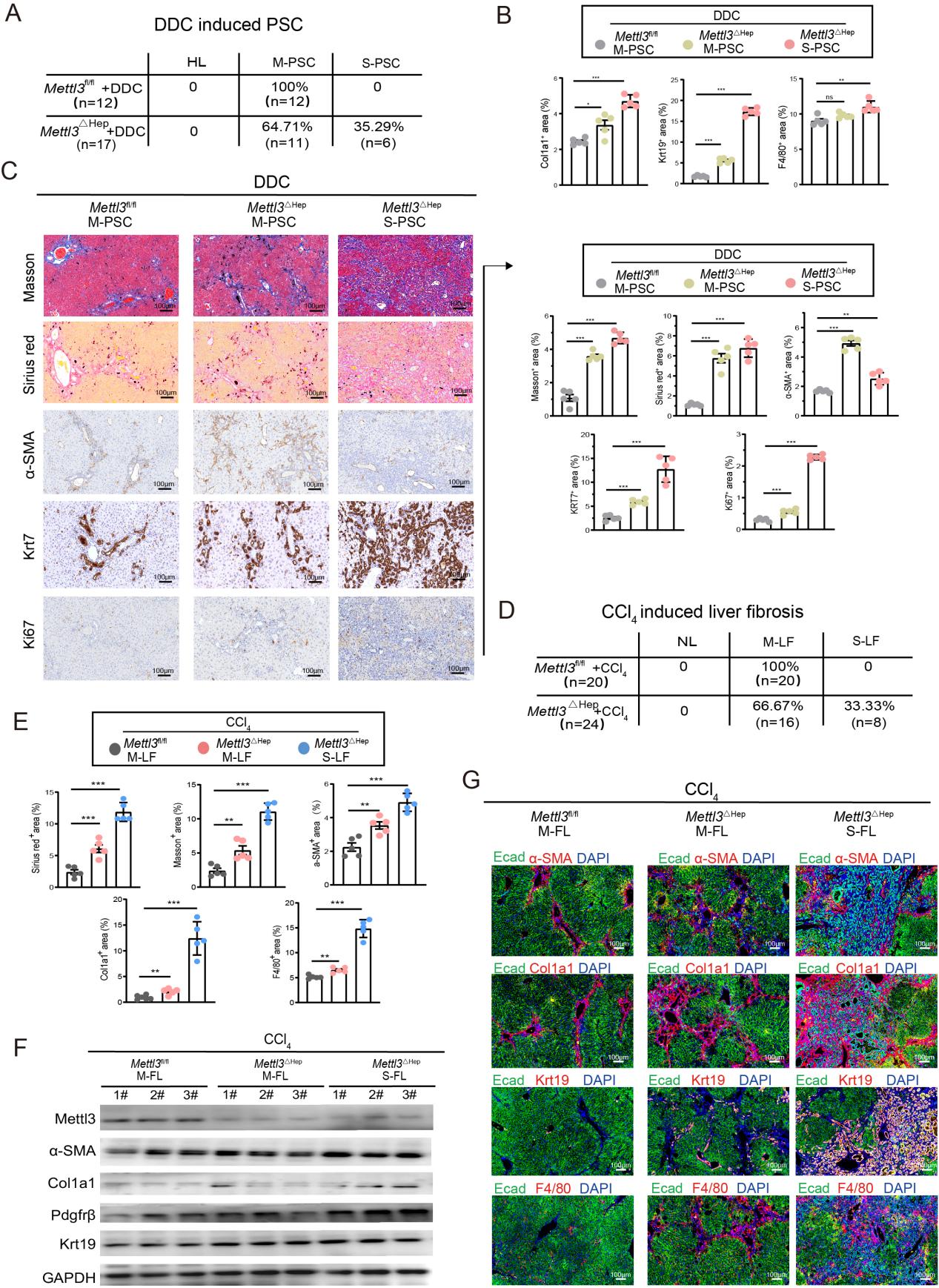
**

Figure S2. Phenotypic characterization of *Mettl3*^△Hep^ in DDC-induced PSC and CCl_4_-induced fibrotic model.

1. Incidence of mild PSC (M-PSC, *n*=11) and severe PSC (S-PSC, *n*=6) in DDC-induced *Mettl3*^△Hep^ and *Mettl3*^fl/fl^ mice (*n=*12). B) Quantification of the positive area percentage of Col1a1, Krt19, and F4/80. C) Representative histology image of Masson's trichrome staining, Sirius red staining, and immunohistochemistry for α-SMA, Krt7, and Ki67 in DDC-induced PSC models of *Mettl3*^△Hep^ and *Mettl3*^fl/fl^ mice. The corresponding quantification is shown on the right side. D) Incidence of mild liver fibrosis (M-LF, *n=*16) and severe liver fibrosis (S-LF, *n=*8) in CCl_4_-treated *Mettl3*^△Hep^ and *Mettl3*^fl/fl^ (*n=*20) mice. E) Quantification of the positive area of Masson's trichrome staining, Sirius red staining, and immunohistochemistry staining of α-SMA, Col1a1, and F4/80. F) Protein levels of Mettl3, α-SMA, Col1a1, Pdgfrβ, and Krt19 in CCl_4_-induced *Mettl3*^fl/fl^ M-LF, *Mettl3*^△Hep^ M-LF, and *Mettl3*^△Hep^ S-LF. G) Representative images of immunofluorescence staining of Ecad (green) with α-SMA (red), Col1a1 (red), Krt19 (red), and F4/80 (red) in CCl_4_-induced liver fibrosis of *Mettl3*^△Hep^ mice and *Mettl3*^fl/fl^ mice. Scale bars: 100 μm, Data represent mean ± SEM; *P < 0.05, **P < 0.01, ***P < 0.001 by two-tailed unpaired Student’s t-test. M-PSC: mild-primary sclerosing cholangitis; S-PSC: severe-primary sclerosing cholangitis; M-LF: mild-liver fibrosis; S-LF: severe-liver fibrosis.


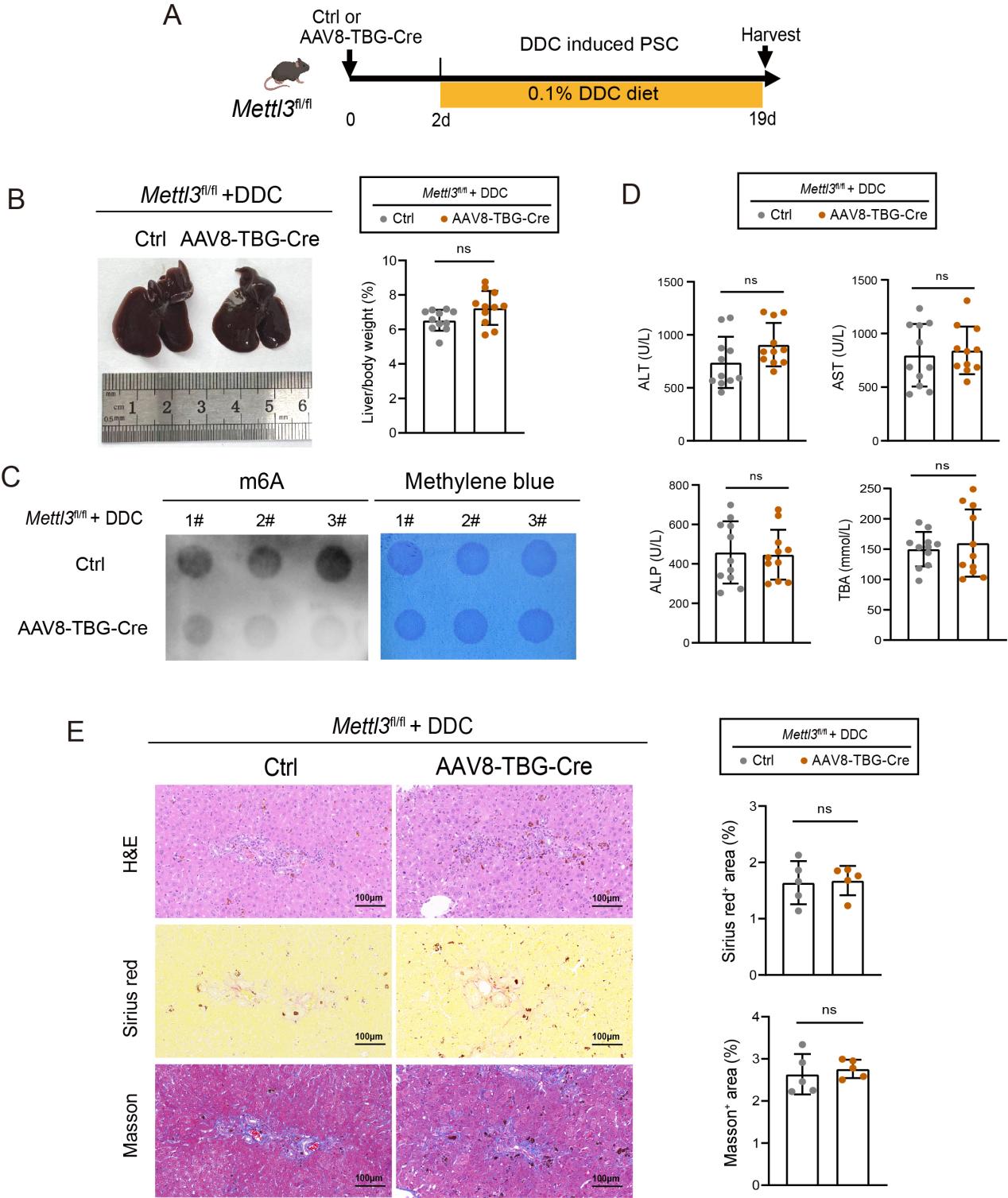
Figure S3. Adult hepatocyte depletion of *Mettl3* did not cause significant differences compared with *Mettl3*^fl/fl^ mice in the DDC-induced PSC model.

1. Experimental design and liver morphology of DDC-induced PSC models in adult *Mettl3*^fl/fl^ control mice (Ctrl, *n=*11), and adult *Mettl3*^fl/fl^ delivered by AAV8-TBG-Cre virus (AAV8-TBG-Cre, *n=*11). B) Representative images of gross liver morphology, liver-to-body weight ratios of Ctrl and AAV8-TBG-Cre group. C) Dot blot analysis of m6A methylation levels in Ctrl and AAV8-TBG-Cre group. D) Serum ALT, AST, ALP, and TBA levels of Ctrl and AAV8-TBG-Cre group following DDC-induced PSC. E) Representative images of histopathology analysis of H&E, Sirius Red, and Masson’s trichrome staining in Ctrl and AAV8-TBG-Cre group following DDC-induced PSC. The corresponding quantification is shown on the right side. Scale bars: 100 μm, Data represent mean ± SEM; *P < 0.05, **P < 0.01, ***P < 0.001 by two-tailed unpaired Student’s t-test.


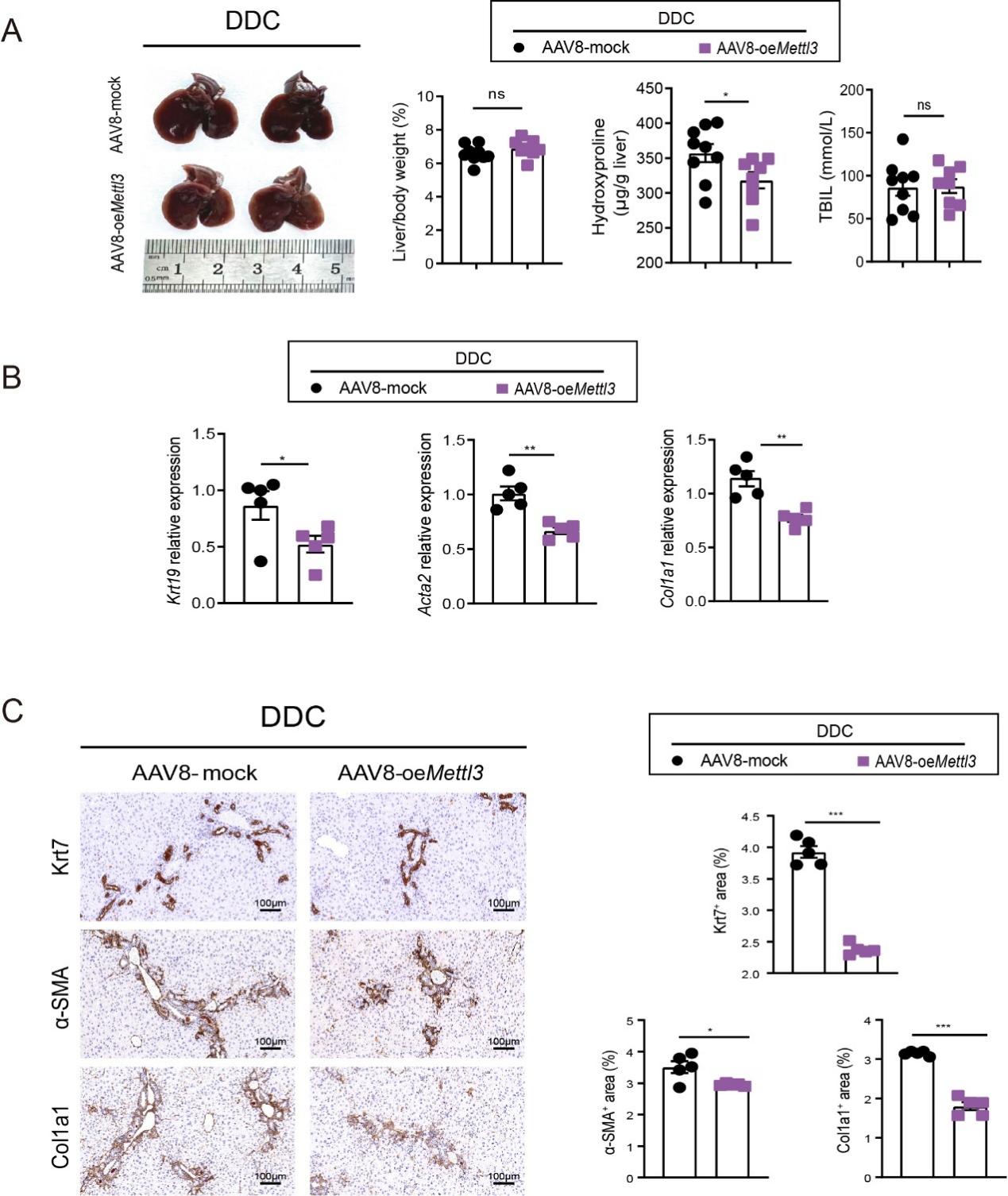


Figure S4. AAV8-mediated *Mettl3* overexpression attenuates pathology in the DDC-induced PSC model.

A) Representative gross liver morphology, liver-to-body weight ratios, hydroxyproline content, serum total bilirubin (TBIL) in AAV8-oe*Mettl3* (*n=*8) compared to AAV8-mock (*n=*9) mice in DDC-induced PSC model. B) Relative gene expression of *Krt19*, *Acta2,*and *Col1a1* in AAV8-oe*Mettl3* (*n=*5) compared to AAV8-mock mice (*n=*5) in DDC-induced PSC model. C) Representative images of IHC staining of Krt7, α-SMA, and Col1a1 in the DDC-treated AAV8-mock and AAV8-oe*Mettl3* mice. The corresponding quantification is shown on the right side. Scale bars: 100 μm. Data represent mean ± SEM; *P < 0.05, **P < 0.01, ***P < 0.001 by two-tailed unpaired Student’s t-test.


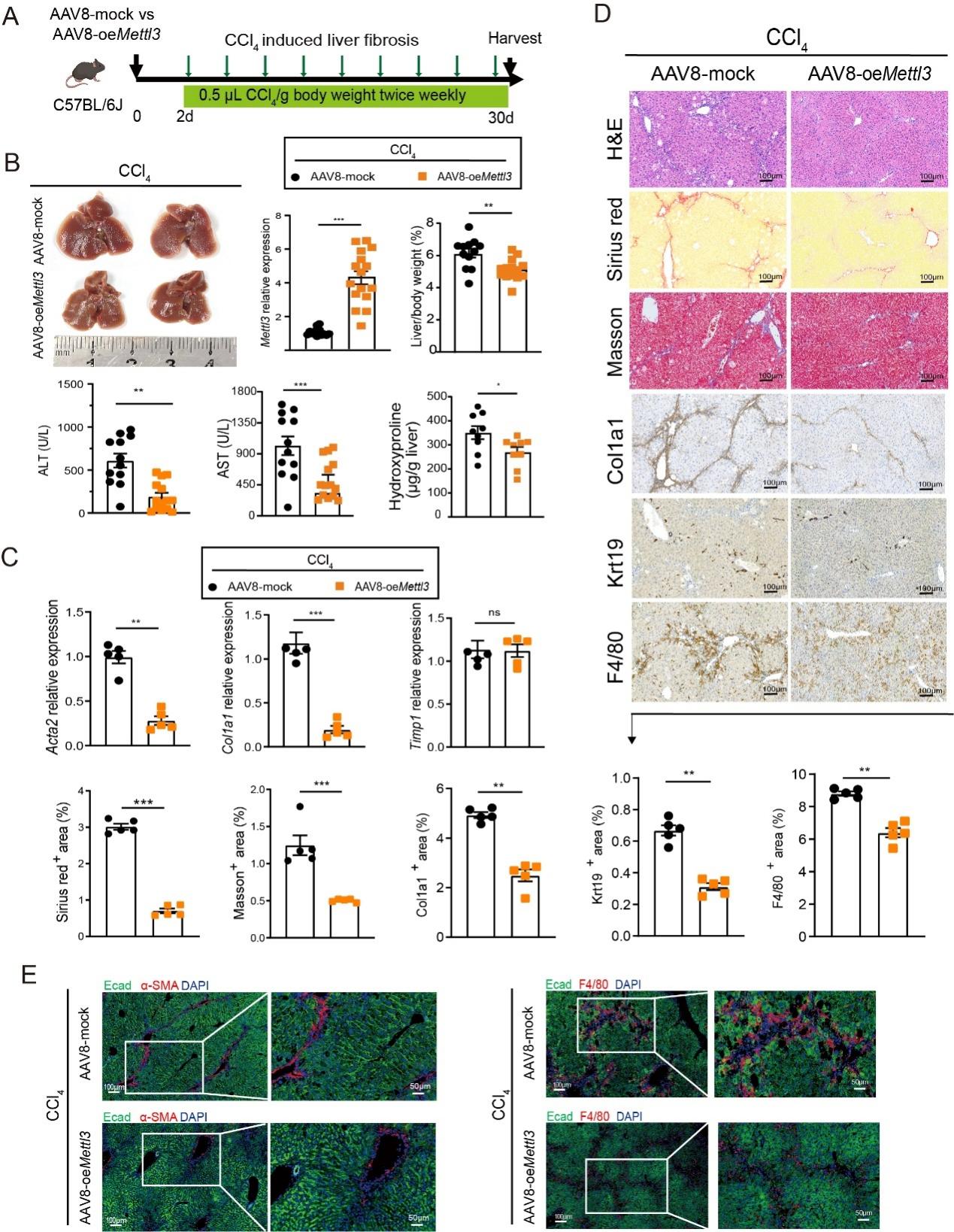


Figure S5. AAV8-mediated *Mettl3* overexpression attenuates pathology in the CCl_4_-induced fibrotic model.

1. Experimental design of CCl_4_-induced liver fibrotic model in AAV8-oe*Mettl3* and AAV8-mock mice. B) Representative gross liver morphology, RT-qPCR validation of *Mettl3* expression, liver-to-body weight ratios, serum biomarkers of ALT and AST, and hydroxyproline content in the CCl_4_-treated AAV8-mock (*n=*12) and AAV8-oe*Mettl3* mice (*n=*14). C) Relative expression of *Acta2,* *Col1a1,* and *Timp1* in the CCl_4_-treated AAV8-mock (*n=*5) and AAV8-oe*Mettl3* mice (*n=*5). D) Representative images of histopathology assessment of H&E, Sirius red, and Masson's trichrome staining, alongside IHC staining of Col1a1, Krt19, and F4/80 in AAV8-mock and AAV8-oe*Mettl3* mice of CCl_4_-induced liver fibrosis, with quantification of the positive area. E) Representative images of immunofluorescence analysis of Ecad (green), α-SMA (red), Col1a1 (red), and F4/80 (red) of AAV8-oe*Mettl3* versus AAV8-mock mice with CCl_4_-induced liver fibrosis. Scale bars: 100 μm. Data represent mean ± SEM; *P < 0.05, **P < 0.01, ***P < 0.001 by two-tailed unpaired Student’s t-test.


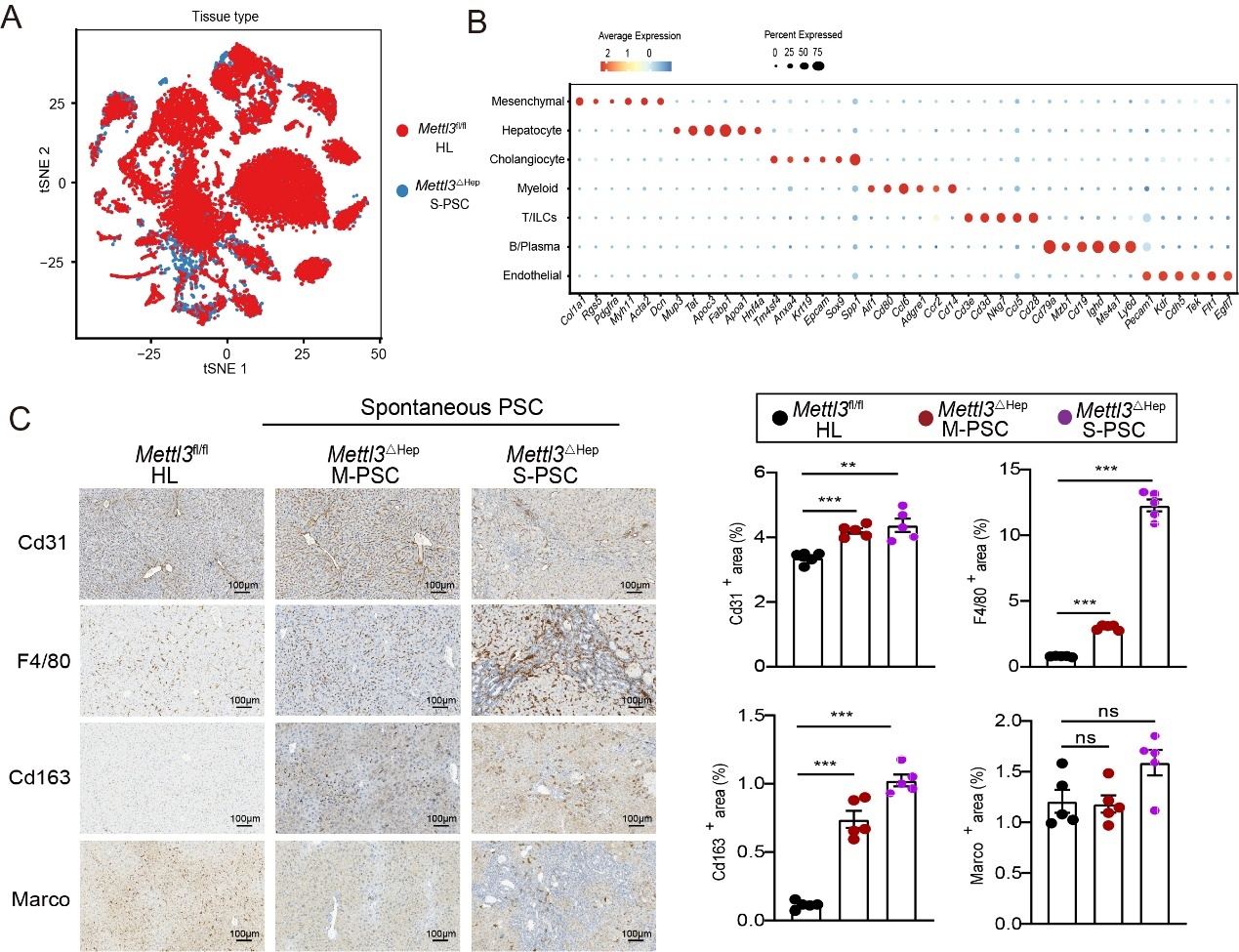


Figure S6. Single-cell RNA sequence analysis of *Mettl3*^△Hep^ S-PSC and *Mettl3*^fl/fl^

A) Dimension reduction plot (t-SNE) showing tissue origin of cells from *Mettl3*^fl/fl^ HL and *Mettl3*^△Hep^ S-PSC. B) Dot plot showing canonical marker genes defining annotated cell clusters. Dot size represents expression percentage; color intensity indicates mean expression. C) Representative images of IHC staining analysis of Cd31, F4/80, Cd163, and Marco in *Mettl3*^fl/fl^ HL, *Mettl3*^△Hep^ M-PSC, and *Mettl3*^△Hep^ S-PSC, with quantification of the positive area. Scale bars: 100 μm. HL: healthy liver; M-PSC: mild-primary sclerosing cholangitis; S-PSC: severe-primary sclerosing cholangitis.


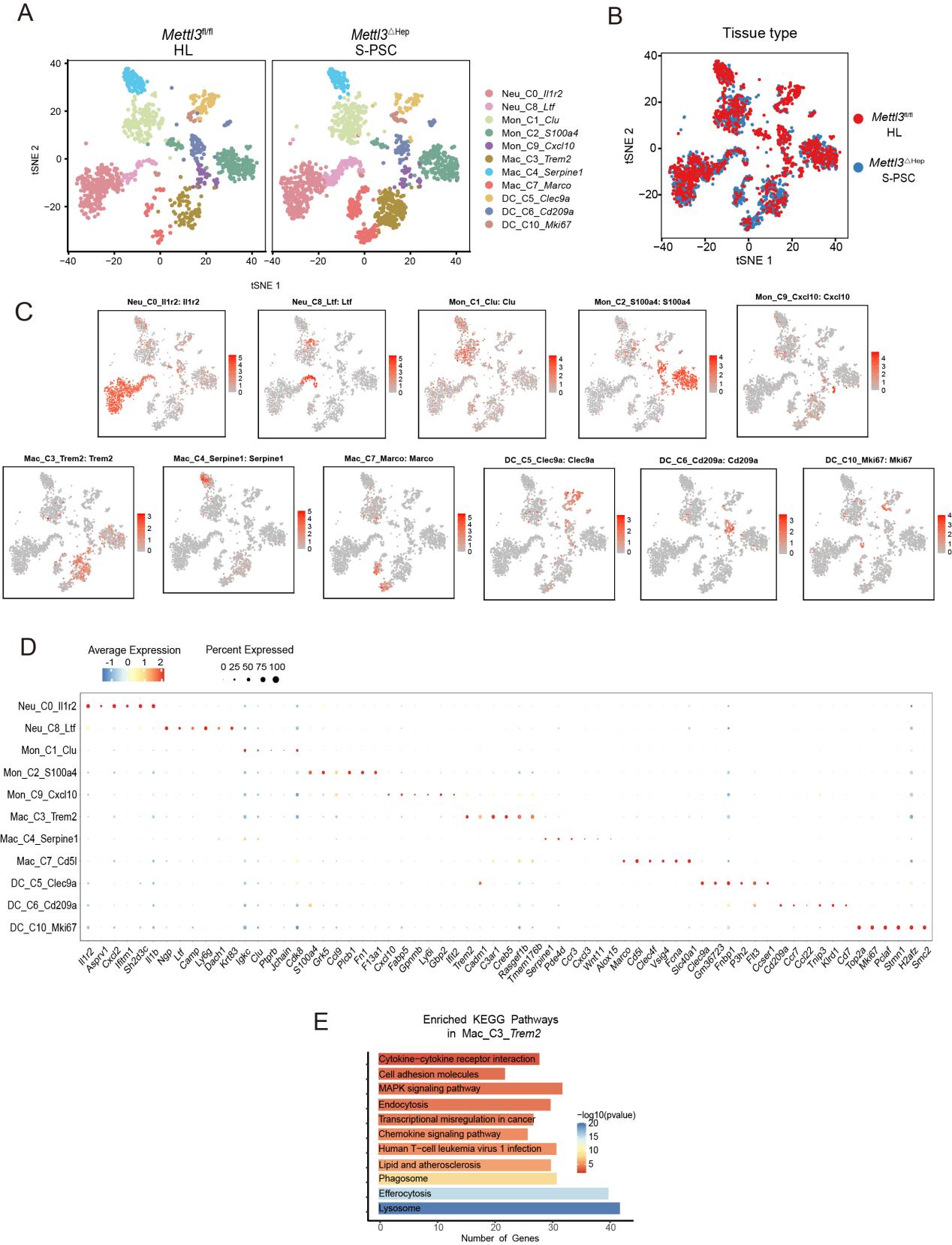


Figure S7. Characteristics of myeloid cell subclusters in *Mettl3*^△Hep^ S-PSC at single-cell resolution.

A) tSNE plot displaying the distributions of 10 myeloid cell subpopulations in *Mettl3*^fl/fl^ HL and *Mettl3*^△Hep^ S-PSC. B) Tissue origin mapping within myeloid clusters in *Mettl3*^fl/fl^ HL and *Mettl3*^△Hep^ S-PSC. C) Feature plots showing expression gradients (gray-to-red) of subtype-specific markers across myeloid subpopulations. D) Dot plot showing marker genes of each myeloid subcluster. E) KEGG pathway enrichment analysis of the Mac_C3_*Trem2* macrophage subset. HL: healthy liver; M-PSC: mild-primary sclerosing cholangitis; S-PSC: severe-primary sclerosing cholangitis.


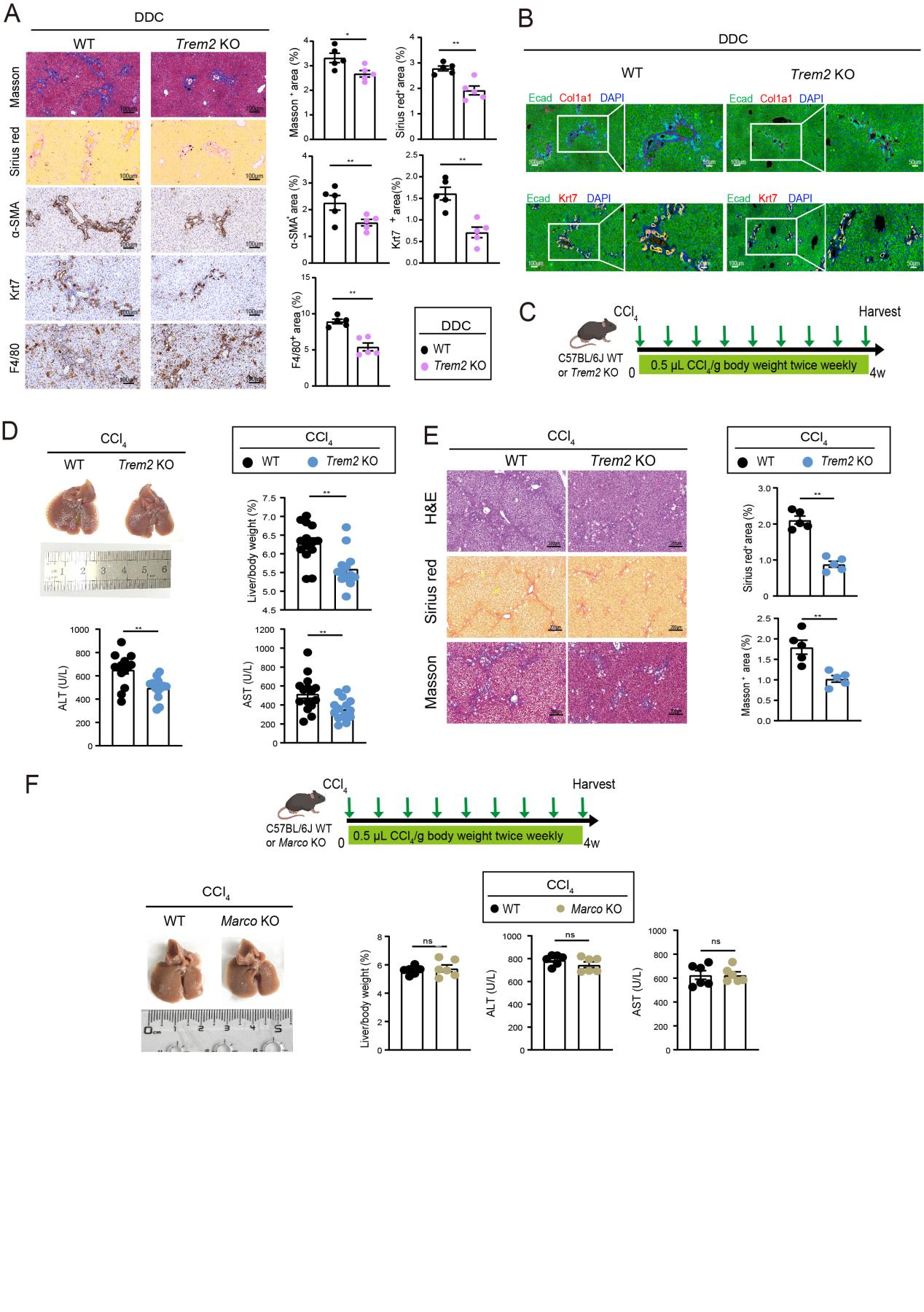
Figure S8. *Trem2*-KO ameliorates DDC-induced PSC and CCl_4_-treated liver fibrosis *in vivo*.

A) Histopathological assessment in DDC-induced PSC models: Masson's trichrome, Sirius red staining, and IHC staining for α-SMA, Krt7, and F4/80 in the livers of wild-type (WT) versus *Trem2*-KO mice, with quantification of the positive area. B) Representative images of immunofluorescence co-staining of Ecad (green) with Col1a1 (red) and Krt7 (red), in the DDC-induced PSC of WT and *Trem2*-KO mice. C) Schematic diagram of intraperitoneal injection of CCl_4_-induced liver fibrosis. D) Gross liver morphology, liver-to-body weight ratio, and serum AST and ALT levels in CCl_4_-induced liver fibrosis of WT (*n=*15) and *Trem2*-KO mice (*n=*15). (E) Representative images of H&E, Masson's trichrome staining, and Sirius red staining of CCl_4_-induced liver fibrosis of WT and *Trem2*-KO mice, with quantification of the positive area. (F) Experimental scheme, gross morphology, liver-to-body weight ratio, and serum AST and ALT levels in CCl_4_ induced-liver fibrosis of WT (*n=*6) and *Marco* KO mice (*n=*6). Scale bars: 100 μm. Data represent mean ± SEM; *P < 0.05, **P < 0.01, ***P < 0.001 by two-tailed unpaired Student’s t-test.


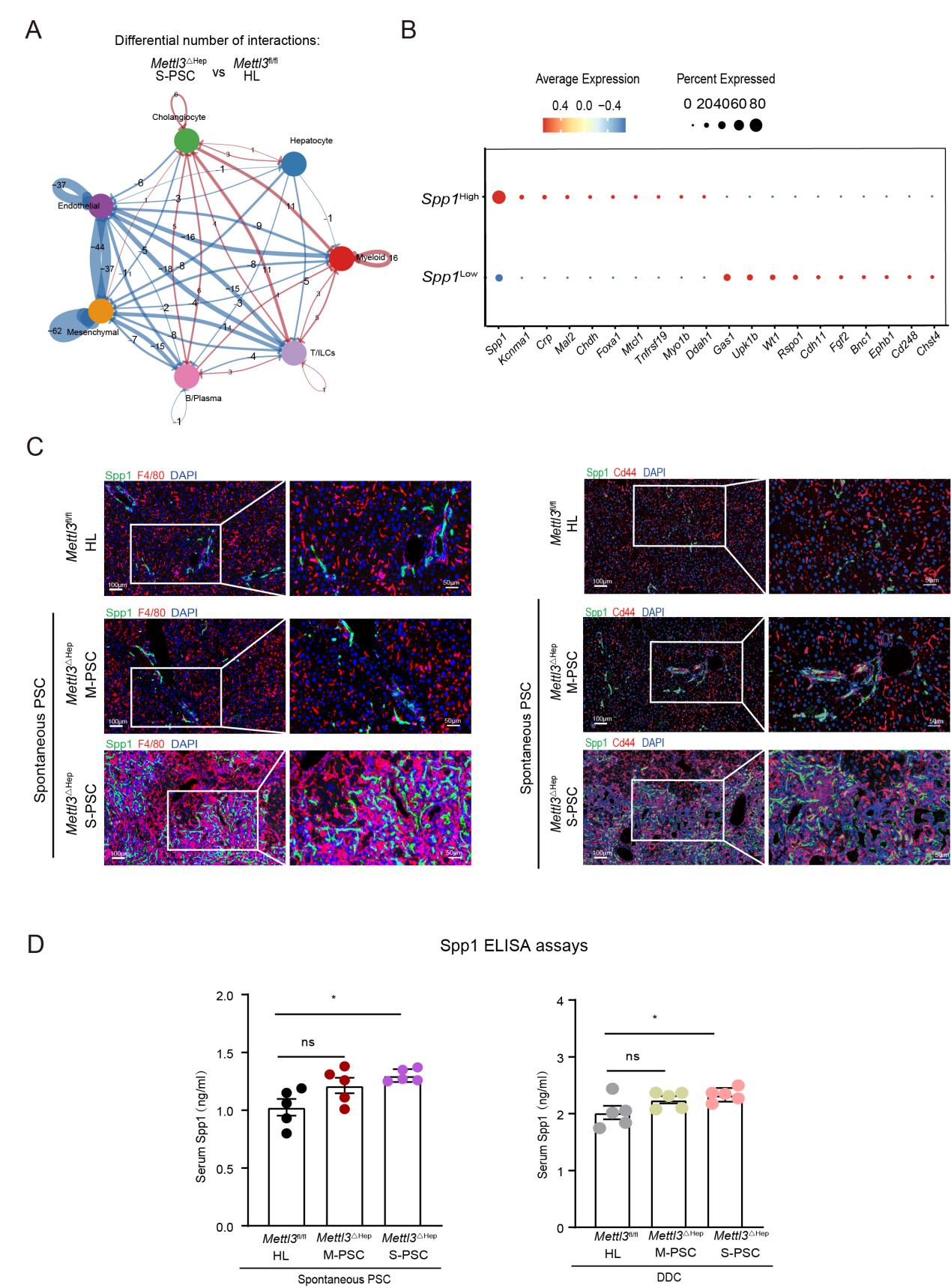


Figure S9. Spp1 was secreted in the cholangiocytes of spontaneous and DDC-induced PSC model of *Mettl3*^△Hep^ mice.

A) Circos plot depicting the differential cell-cell interaction networks among major cell types in *Mettl3*^△Hep^ S-PSC versus *Mettl3* ^fl/fl^ HL livers. B) Dot blot showing the marker gene expression of Cho_*Spp1*^High^ and Cho_*Spp1*^Low^ subclusters. C) Representative images of immunofluorescence co-staining of Spp1 (green) and F4/80 (red) (left panel), Spp1 (green) and Cd44 (red) (right panel), in the liver sections of *Mettl3*^fl/fl^ HL, *Mettl3*^△Hep^ M-PSC, and *Mettl3*^△Hep^ S-PSC. Scale bars: 100 μm; insert, 50 μm. D) ELISA assays of Spp1 secretion in the serum of spontaneous and DDC induced PSC of *Mettl3*^△Hep^ S-PSC (*n=*5), *Mettl3*^△Hep^ M-PSC (*n=*5), and *Mettl3* ^fl/fl^ (*n=*5). Data represent mean ± SEM; *P < 0.05, **P < 0.01, ***P < 0.001 by two-tailed unpaired Student’s t-test. HL: healthy liver; M-PSC: mild-primary sclerosing cholangitis; S-PSC: severe-primary sclerosing cholangitis.


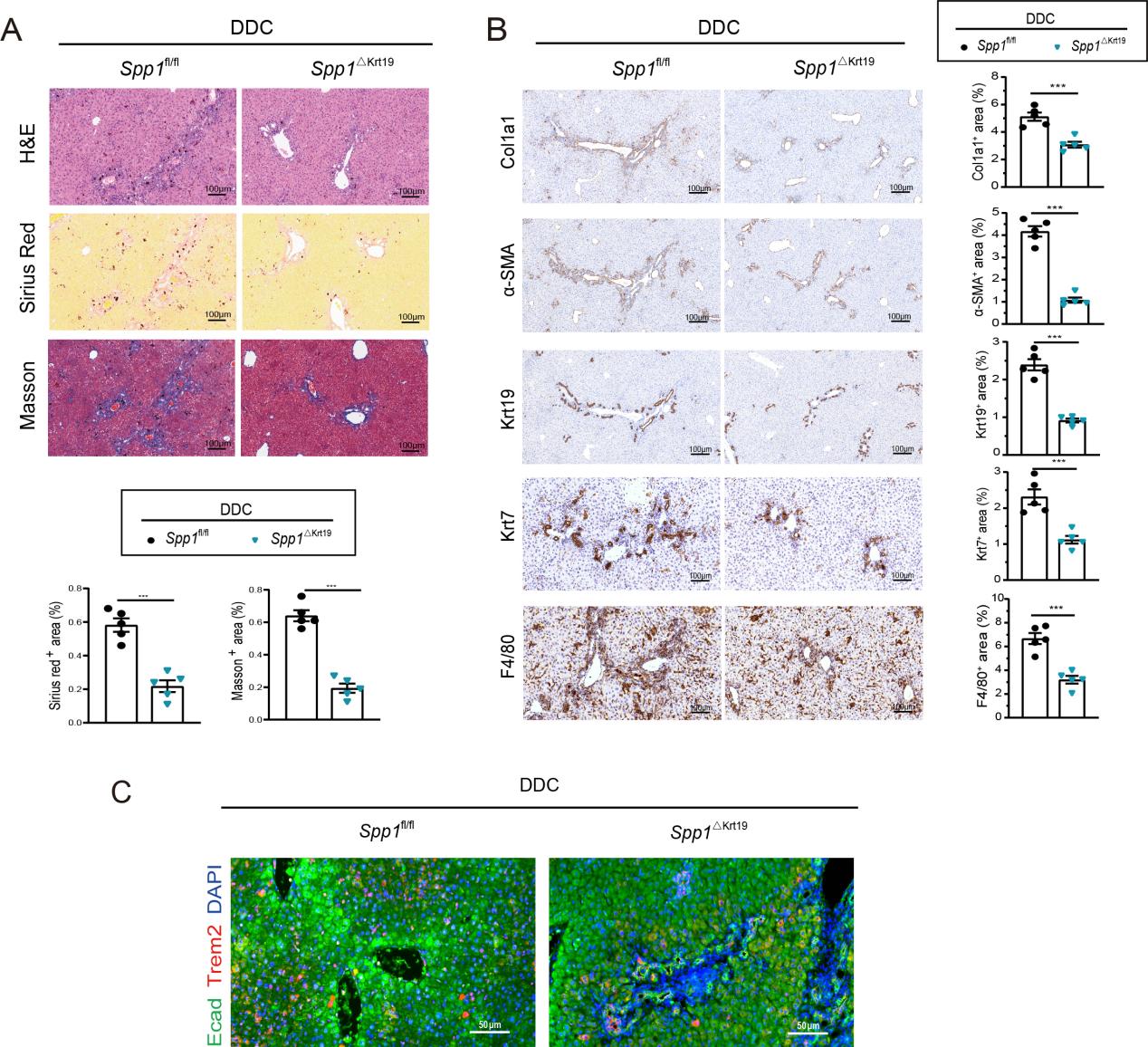


Figure S10. Cholangiocyte specific deletion of *Spp1* repressed DDC-induced PSC progression.

A) Representative images of H&E, Sirius red, and Masson's trichrome staining in the DDC-induced PSC of *Spp1*^fl/fl^ and *Spp1*^△krt19^ mice, with quantification of the positive area. B) Representative images of IHC staining for ductal markers (Krt19, Krt7), fibrotic markers (Col1a1, α-SMA), and macrophage (F4/80) of DDC-treated PSC of *Spp1*^fl/fl^ and *Spp1*^△krt19^ mice, with quantification of the positive area. C) Representative images of immunofluorescence staining of Ecad (green) and Trem2 (red) in the liver sections of DDC-induced PSC of *Spp1*^fl/fl^ and *Spp1*^△krt19^ mice. Data represent mean ± SEM; *P < 0.05, **P < 0.01, ***P < 0.001 by two-tailed unpaired Student’s t-test. Scale bars: 100 μm (A, B) or 50 μm (C).


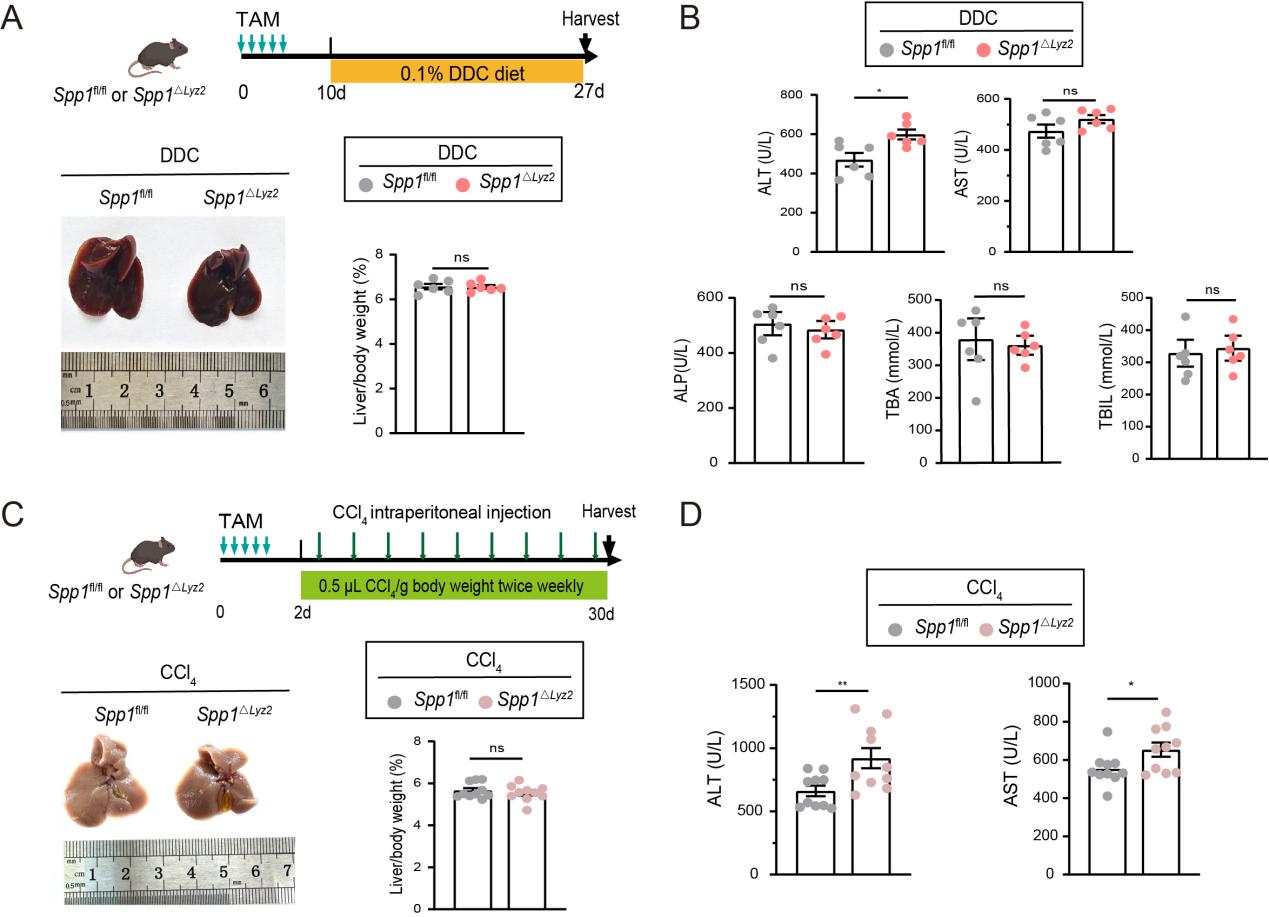


Figure S11. Myeloid cell specific deletion of *Spp1* in the DDC-induced PSC and CCl_4_-treated liver fibrosis.

A) Experimental design, liver morphology, liver-to-body weight ratios of DDC-induced PSC models in *Spp1^△Lyz2^* (*n=*6) compared with WT mice (*n=*6). B) Serum biochemical markers (ALT, AST, ALP, TBA, and TIBL) in DDC induced PSC of *Spp1^△Lyz2^* mice (*n=*6) versus *WT* controls (*n=*6). Data are represented as mean± SEM. C) Experimental design, gross liver morphology, liver-to-body weight ratios of CCl_4_-treated liver fibrosis in *Spp1^△Lyz2^* (*n=*10) compared with WT mice (*n=*10). D) Serum biochemical markers (ALT, AST) in CCl_4_-treated liver fibrosis of *Spp1^△Lyz2^* mice (*n=*10) versus *WT* controls (*n=*10). Data represent mean ± SEM; *P < 0.05, **P < 0.01, ***P < 0.001 by two-tailed unpaired Student’s t-test.


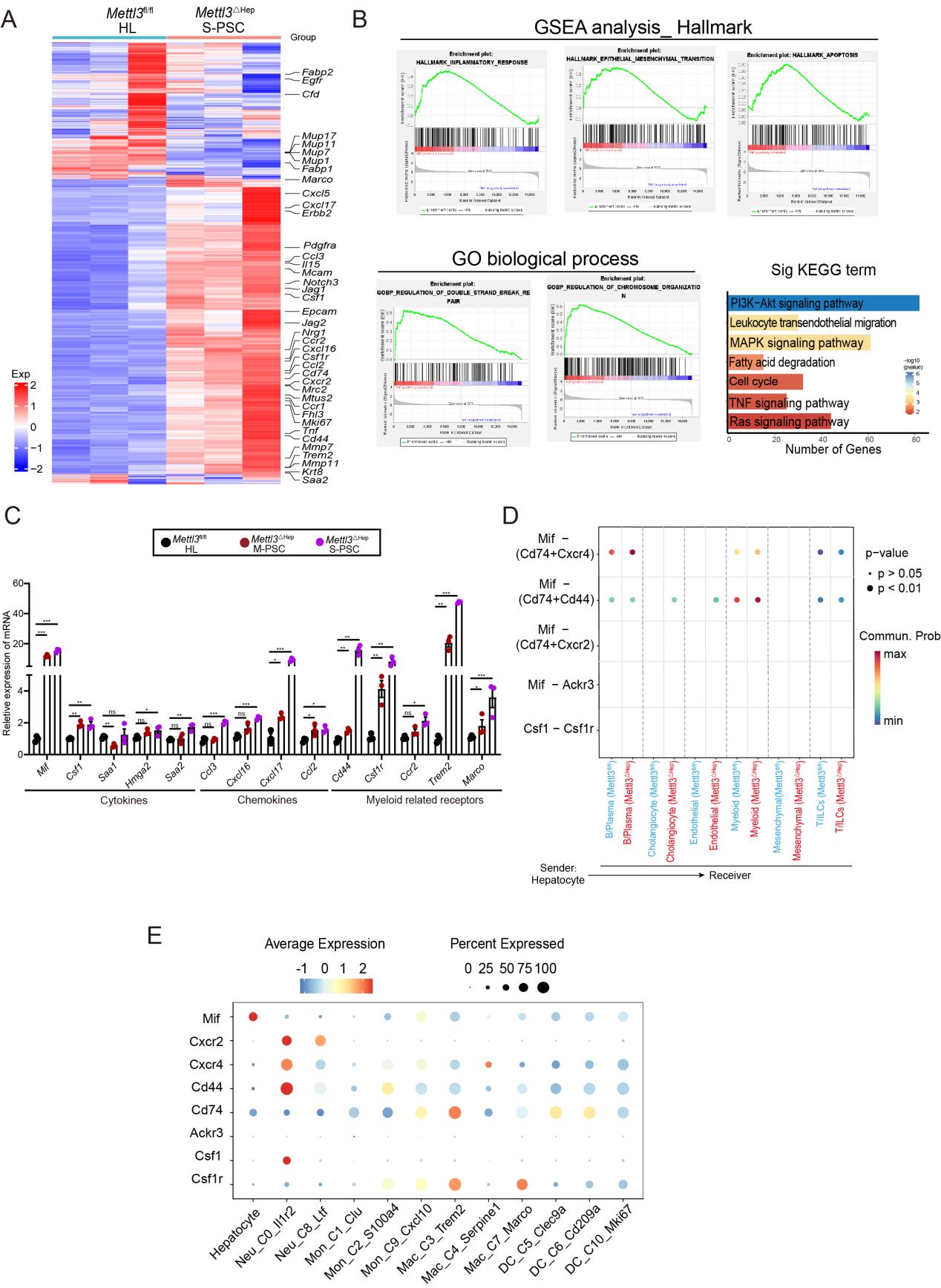


Figure.S12. Integrated bulk and single cell-RNA sequencing revealed a hepatocyte Mif-Cd44 axis that drives myeloid cell recruitment in the *Mettl3*^△Hep^ S-PSC.

1. Heatmap depicting significantly differentially expressed genes in the bulk RNA sequence of *Mettl3*^△Hep^ S-PSC (*n=*3) compared to *Mettl3*^fl/fl^ mouse liver tissues (*n=*3). B) Gene set enrichment analysis (GSEA), Gene Ontology (GO), and KEGG pathway analysis of the bulk RNA-sequence data form *Mettl3*^△Hep^ S-PSC compared to *Mettl3*^fl/fl^ mouse liver tissues. C) RT-qPCR validation of selected cytokines, chemokines, and myeloid receptor genes identified by the bulk RNA-seq in *Mettl3*^△Hep^ S-PSC compared to *Mettl3*^fl/fl^ livers. D) Enrichment of *Mif* and *Csf1*-related ligand-receptor pairs between hepatocytes and major cell types in *Mettl3*^ΔHep^ S-PSC compared to *Mettl3* ^fl/fl^controls. E) Dot plot demonstrating the expression levels of *Mif* and *Csf1-*related ligand-receptor pair across hepatocytes and myeloid cell subtypes.


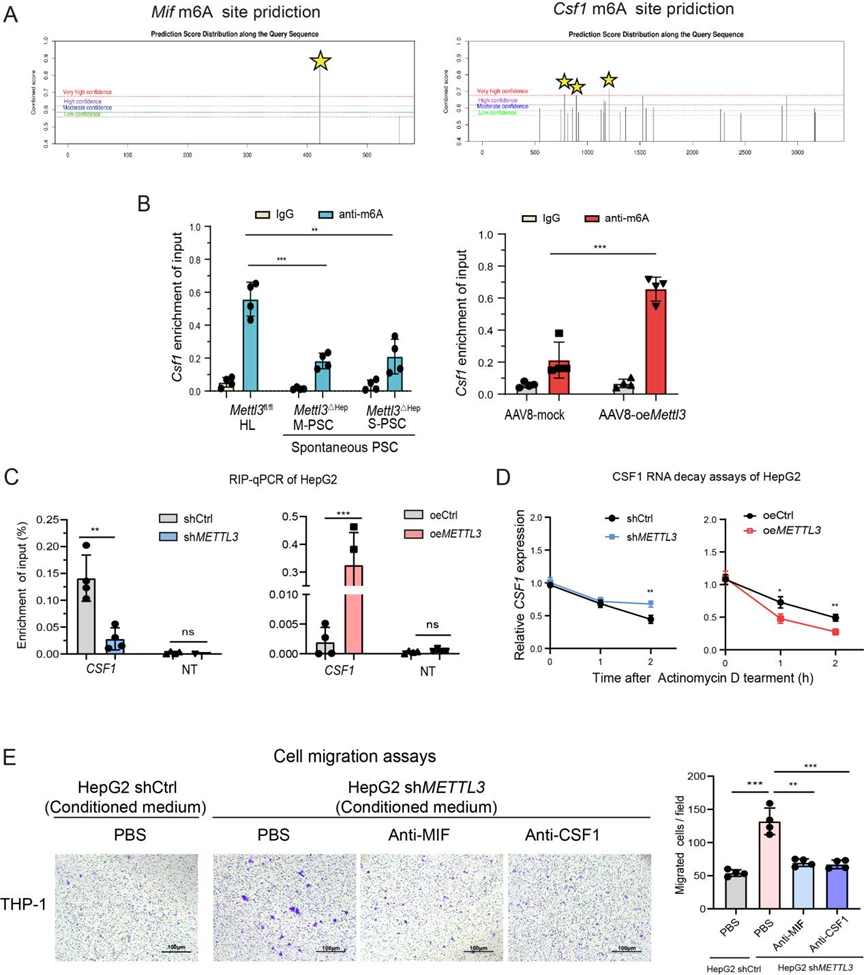


Figure S13. Mettl3 stabilizes *Csf1* mRNAs through m⁶A-dependent mechanisms in hepatocytes.

A) SRAMP database prediction of high-confidence m⁶A modification sites in *Mif* and *Csf1* mRNAs (sequence motifs shown with position coordinates). B) In vivo m6A-RIP-qPCR quantification of *Csf1* mRNA enrichment in liver tissues from *Mettl3*^fl/fl^ HL, *Mettl3*^△Hep^ M-PSC, and *Mettl3*^△Hep^ S-PSC; AAV8-mock, and AAV8-oe*Mettl3* mice. C) In vitro validation of m6A-RIP-qPCR quantification of *CSF1* mRNA enrichment in METTL3-modulated hepatocytes, in HepG2 cells with *METTL3* knockdown (sh*METTL3*) or overexpression (oe*METTL3*). D) mRNA stability assay of *CSF1* transcript half-life measured by RT-qPCR after actinomycin D treatment (5 μg/mL) in HepG2 cells with *METTL3* knockdown (sh*METTL3*) or overexpression (oe*METTL3*). E) THP-1 Transwell migration assays using conditioned medium from HepG2 sh*METTL3* with or without neutralization antibodies against MIF or CSF1. Data represent mean ± SEM; *P < 0.05, **P < 0.01, ***P < 0.001 by two-tailed unpaired Student’s t-test.


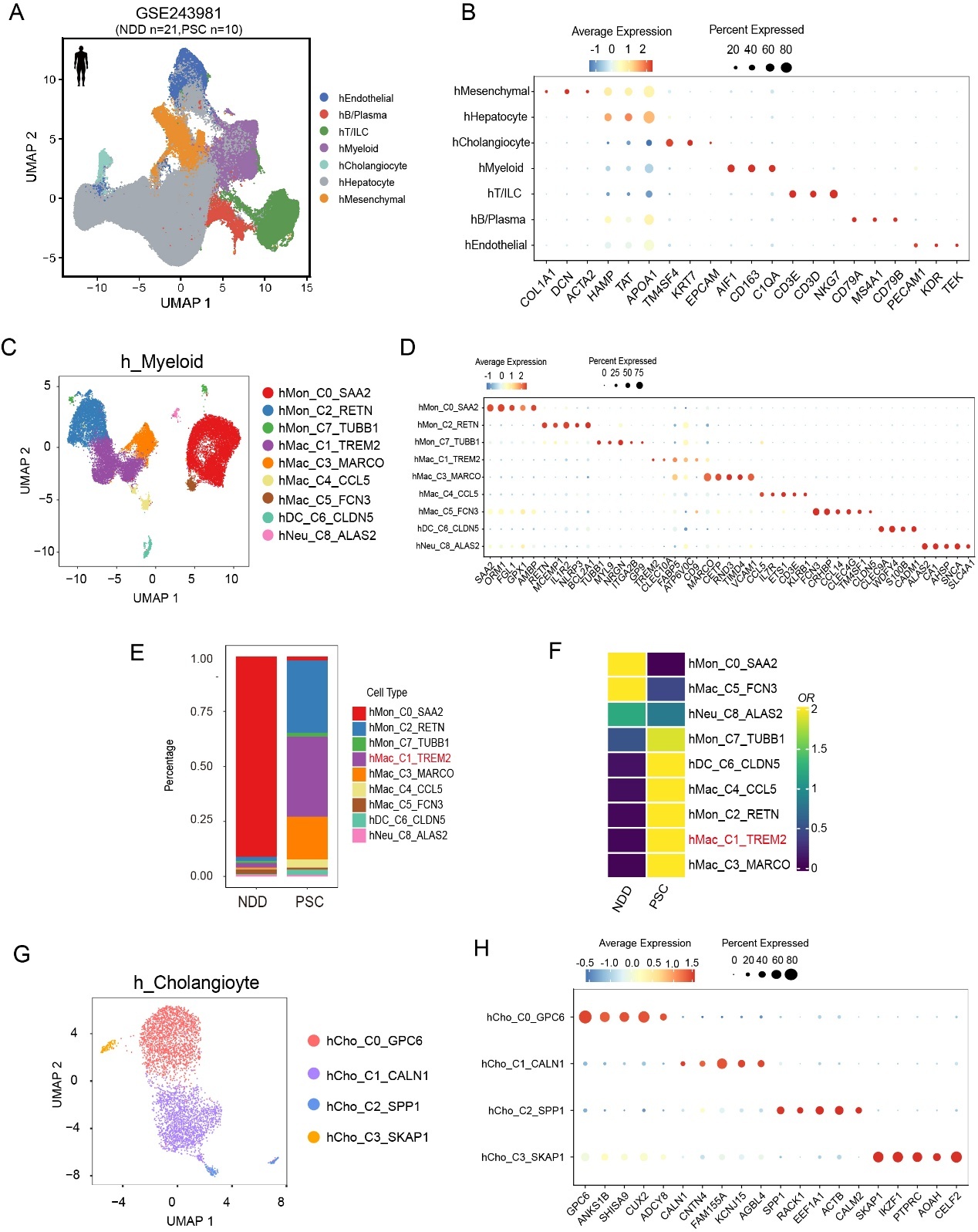


Figure S14. Single-cell transcriptomic analysis of human PSC.

1. UMAP visualization of cell types in human PSC (*n=*10) and non-disease donor (NDD) controls (*n=*21). B) Expression patterns of canonical markers used for annotation across major cell types. C) UMAP visualization of macrophage subpopulations in NDD controls and human PSC samples. D) Top five marker genes of each macrophage subpopulations. E) Relative abundance of each myeloid cell subpopulation in the NDD controls versus PSC. F) Odds ratios (OR) indicating the enrichment of myeloid subpopulations in human PSC. G) UMAP visualization depicting the annotation of cholangiocyte subpopulations in the NDD controls and PSC samples. H) The top five marker genes for each cholangiocyte subpopulation.


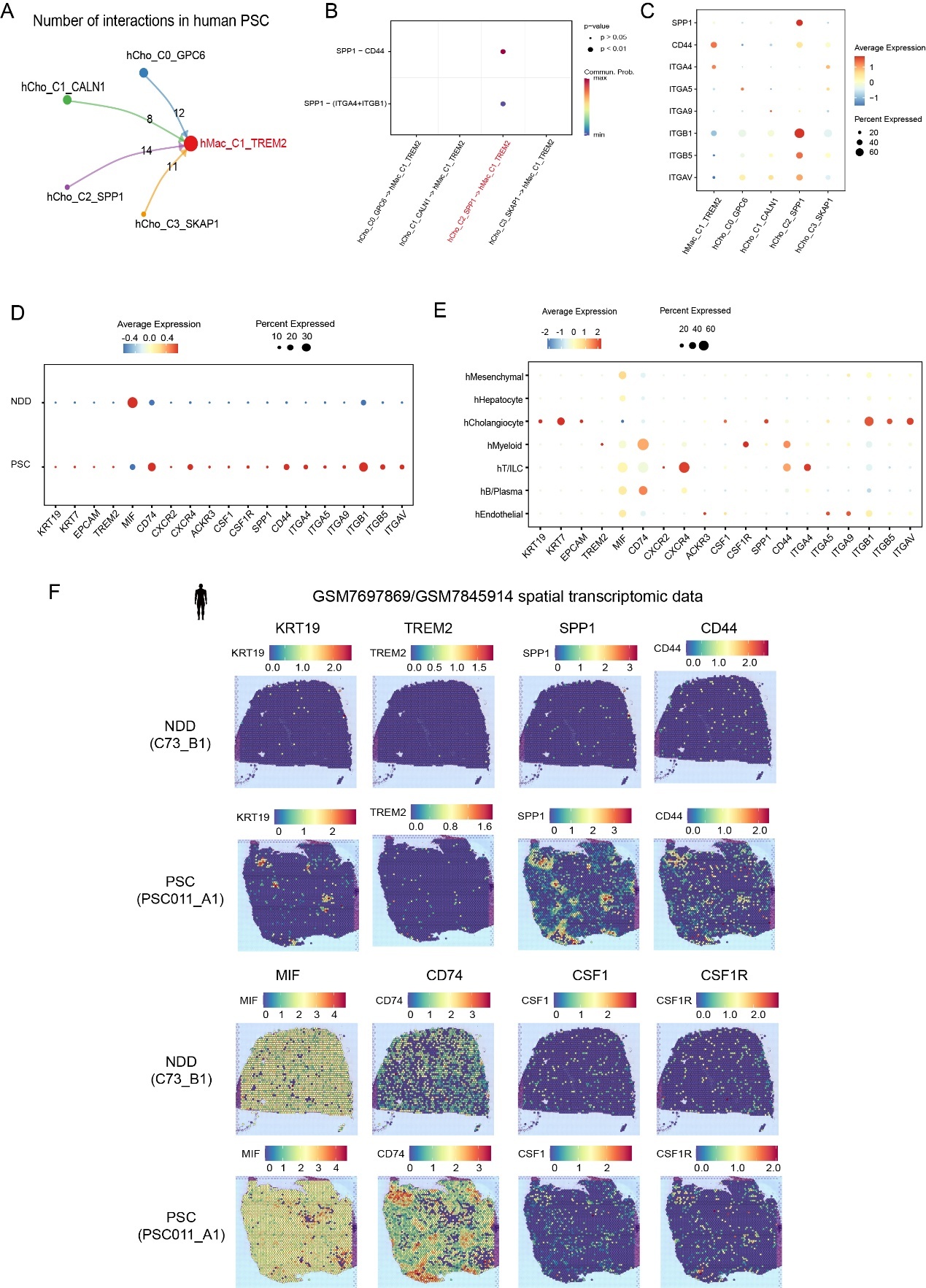


Figure S15. Cell-cell interaction and spatial analysis of the SPP1-CD44 axis in human PSC.

A) Cell interaction network between the hMac_C1_TREM2 and cholangiocyte subclusters in human PSC. B) Validation of the enrichment of the SPP1-CD44 ligand-receptor pair between cholangiocyte subpopulations and hMac_C1_TREM2 in human PSC. C) Dot plot depicting the expression patterns of SPP1 and its related receptors in cholangiocyte subpopulations and hMac_C1_TREM2. D, E) Dot plot showing key gene expression of the interaction between Trem2^+^ macrophage subcluster and Spp1^+^ cholangiocyte in the NDD controls and human PSC samples (D) or major cell types (E). F) Spatial localization of key genes in the NDD controls and PSC patients.


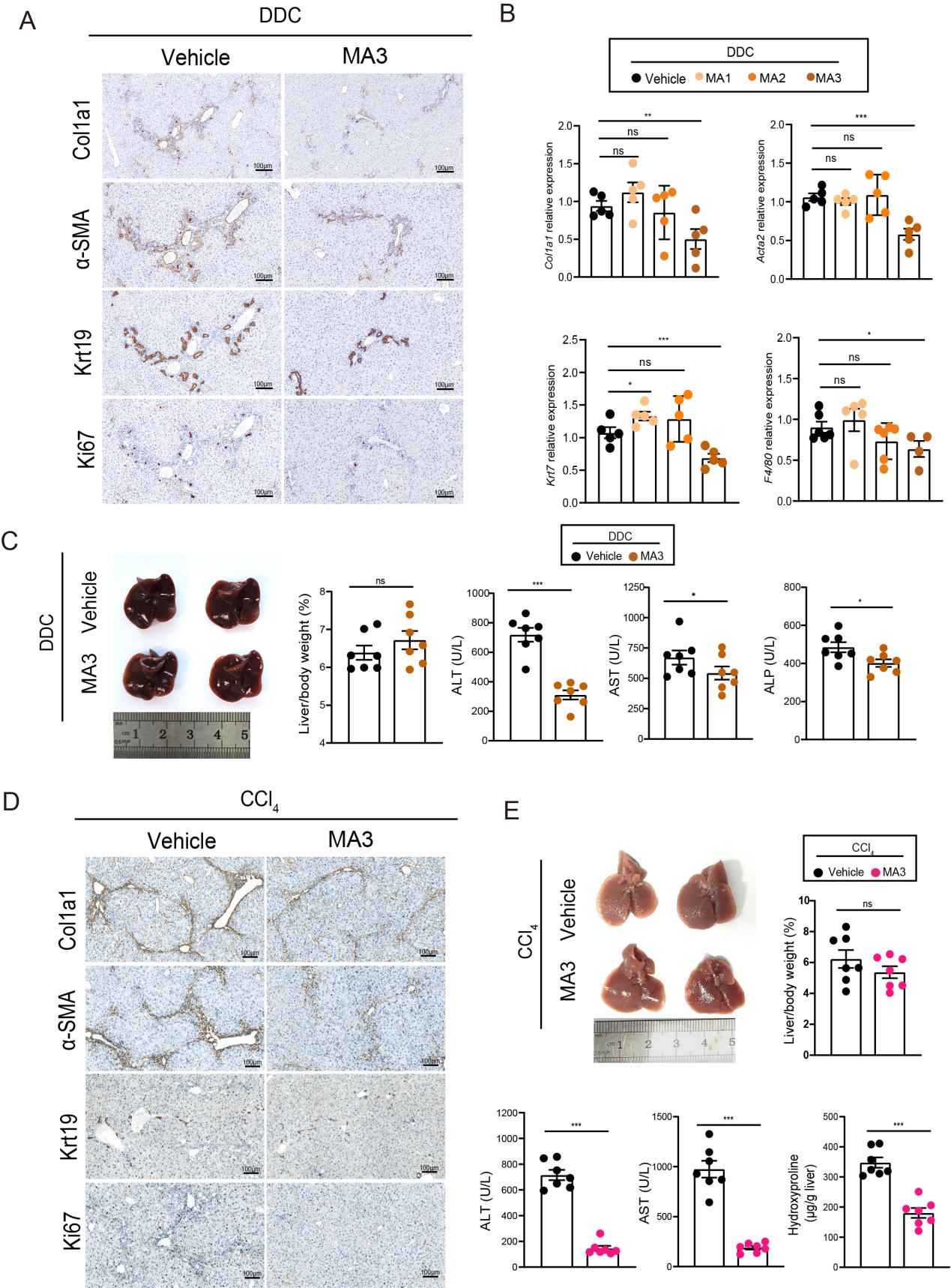


Figure S16. Therapeutic efficacy of Mettl3 agonist MA3 in the murine PSC and liver fibrotic models.

A) Representative IHC images of liver sections stained for the fibrotic markers α-SMA and Col1a1, ductular reaction marker Krt19, and proliferation marker Ki67, in DDC-treated C57BL/6J mice, administered vehicle versus MA3, respectively. B) RT-qPCR analysis of *Col1a1, Acta2, Krt7*, and *F4/80* in DDC-induced PSC models treated with vehicle, MA1, MA2, and MA3, respectively. C) Representative gross liver morphology, liver-to-body weight ratios, and serum levels of ALT, AST, and ALP in DDC-induced PSC models when treated with vehicle or MA3, respectively. D) Representative images of IHC analysis for α-SMA, Col1a1, Krt19, and Ki67 in liver sections from CCl_4_-induced liver fibrotic mice treated with vehicle or MA3, respectively. E) Comparison of liver macroscopic appearance, liver to body weight ratios, serum AST and ALT levels, and hepatic hydroxyproline content in CCl4-induced liver fibrotic mice treated with vehicle or MA3, respectively. Scale bars: 100 μm (A, D). Data represent mean ± SEM; *P < 0.05, **P < 0.01, ***P < 0.001 by two-tailed unpaired Student’s t-test. MA: Mettl3 agonist.


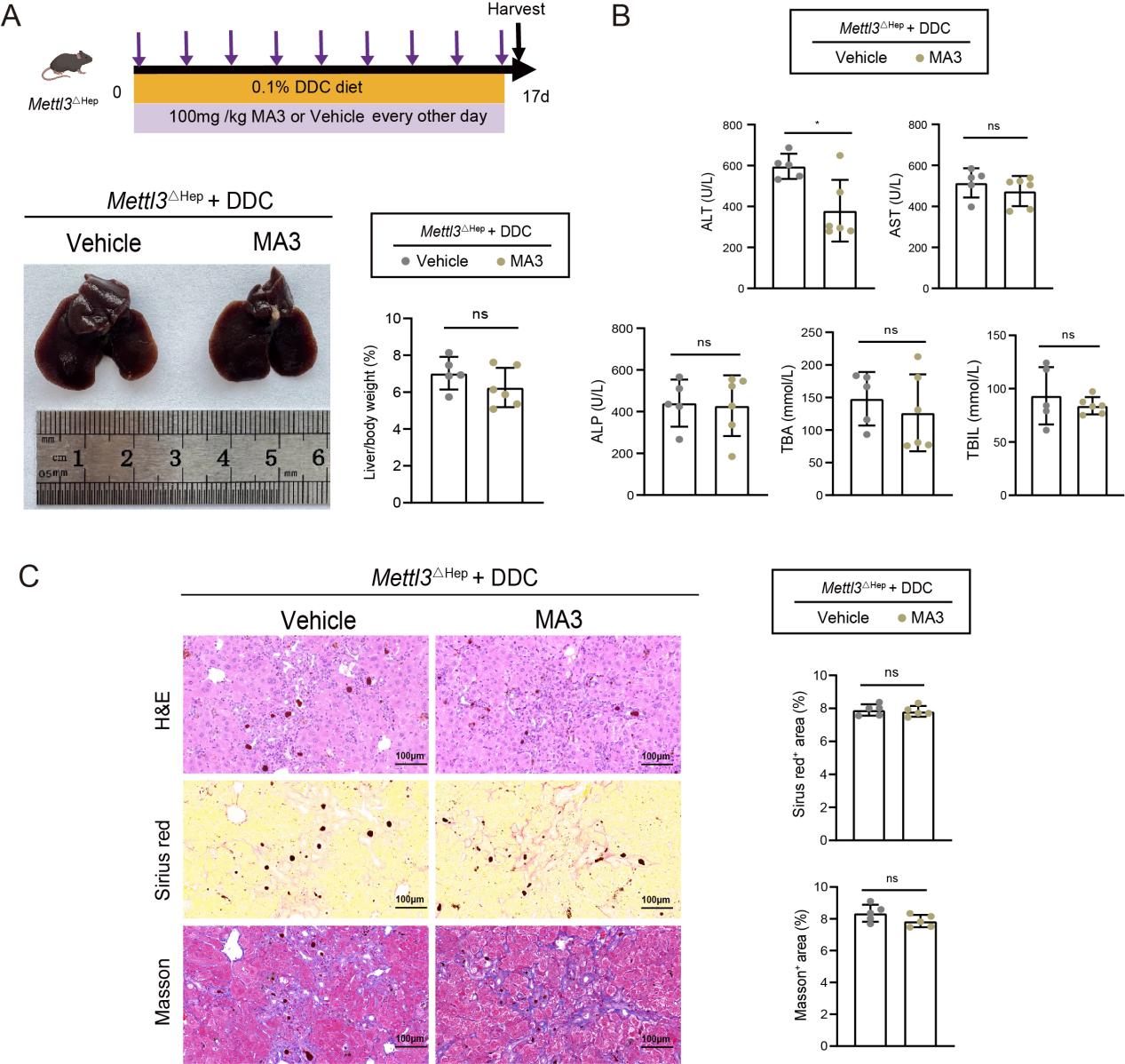
Figure S17

Figure S17. MA3 treatment failed to ameliorate DDC-induced PSC in the Mettl3^∆Hep^ mice.

A) Experimental design, representative gross liver morphology, and liver-to-body weight ratios in DDC-induced PSC models of Mettl3^∆Hep^ mice when treated with vehicle (*n=*5) or MA3(*n=*6), respectively. B) Serum levels of ALT, AST, ALP, TBA, and TBIL in DDC-induced PSC of Mettl3^∆Hep^ mice when treated with vehicle (*n=*5) or MA3(*n=*6), respectively. C) Representative images of H&E, Sirius red, and Masson's trichrome staining in the DDC-induced PSC of Mettl3^∆Hep^ mice when treated with vehicle or MA3, with quantification of the positive area. Scale bars: 100 μm. Data represent mean ± SEM; *P < 0.05, **P < 0.01, ***P < 0.001 by two-tailed unpaired Student’s t-test.

**Table S1. Primer Sequences used in mouse PCR genotyping.**

| Mouse in use | Sequence of genotyping primers（5'→3'） | Supplier |
| --- | --- | --- |
| *Alb*-CreERT2 | Forward: GGCCTCCAAGTCTTGACAGTAGATT  Reverse: TTGTGGGTCTTCCACCTTTCTTC | GemPharmatech Co. Ltd. |
| *Alb*-Cre | Forward: GCCTGCATTACCGGTCGATGC  Reverse: CAGGGTGTTATAAGCAATCCC | Shanghai Model Organisms Center,Inc. |
| *Krt19*-CreERT2 | Forward: CAGAATCGCCAGGAATTGAC  Reverse1: CGGAAAACACCCCCTGA  Reverse2: AGGCAAATTTTGGTGTACGG | Shanghai Model Organisms Center,Inc. |
| *Mettl3* ^flox/flox^ | Forward: AACCATCCAAACCTTTACTGTGC  Reverse: TAAACATAAACACCAGGCCCTTG | Cyagen Biotechnology Co. Ltd. |
| *Rosa26*-lsl-*Mettl3* | Forward1: CCTGGCACCCGCAAGATT  Reverse1: GCAAAGGGTCGCTACAGACG  Forward2: ATAGTTATGAGGAGTGAGGTGGAC  Reverse2: TATGCAGATGGTTTAAAGACAGGT | Shanghai Model Organisms Center,Inc. |
| *Spp1*^flox/flox^ | Forward: GGTCCCTGTGTGATAACACAGACTC  Reverse: CTCTCCACCTCTCATCCTTAGCAAG | GemPharmatech Co. Ltd. |
| *Trem2* KO | Forward1: GATGTCTTAAATAGAGCCAGAGGG Forward2: ACATTCAGGGATTCCACAGGTC  Reverse1: GGAAGGTGGTAGGCTAGAGGTGAC | Cyagen Biotechnology Co. Ltd. |
| *Marco* KO | Forward1: TCTGAATTCCTTTTGGCCTATGTC  Reverse1: TTTTACGGCCCAGCTAGTTCTATT  Reverse2: AAATCTCCAGAGCCACATAAAGGC | Cyagen Biotechnology Co. Ltd. |
| C57BL/6J | - | Beijing Vital River Laboratory Animal Technology Co.Ltd. |

**Table S2. Primer sequences for quantitative real-time PCR (qPCR).**

| Name | Sequence |
| --- | --- |
| *Gapdh* | Forward : AGGTCGGTGTGAACGGATTTG  Reverse : GGGGTCGTTGATGGCAACA |
| *Mettl3* | Forward : CTGGGCACTTGGATTTAAGGAA  Reverse : TGAGAGGTGGTGTAGCAACTT |
| *Acta2* | Forward : CCCAGACATCAGGGAGTAATGG  Reverse: TCTATCGGATACTTCAGCGTCA |
| *Col1a1* | Forward : GCTCCTCTTAGGGGCCACT  Reverse: ATTGGGGACCCTTAGGCCAT |
| *Col1a2* | Forward : TCGTGCCTAGCAACATGCC  Reverse : TTTGTCAGAATACTGAGCAGCAA |
| *Des* | Forward : CCTGGAGCGCAGAATCGAAT  Reverse : TGAGTCAAGTCTGAAACCTTGGA |
| *Mmp3* | Forward : GGCCTGGAACAGTCTTGGC  Reverse : TGTCCATCGTTCATCATCGTCA |
| *Vim* | Forward : CGTCCACACGCACCTACAG  Reverse : GGGGGATGAGGAATAGAGGCT |
| *Timp1* | Forward : CGAGACCACCTTATACCAGCG  Reverse : ATGACTGGGGTGTAGGCGTA |
| *Krt19* | Forward: GTTCAGTACGCATTGGGTCAG  Reverse: GAGGACGAGGTCACGAAGC |
| *Mif* | Forward : GAACCGCAACTACAGTAAGCTGC  Reverse : ACGTTGGCAGCGTTCATGTCGT |
| *Csf1* | Forward : GCCTCCTGTTCTACAAGTGGAAG  Reverse : ACTGGCAGTTCCACCTGTCTGT |
| *Saa1* | Forward : GGAGTCTGGGCTGCTGAGAAAA  Reverse : TGTCTGTTGGCTTCCTGGTCAG |
| *Hmga2* | Forward : AGAGGAAGACCCAAAGGCAGCA  Reverse : GAGCAGGCTTCTTCTGAACGAC |
| *Saa2* | Forward : GGAGTCTGGGCTGCTGAGAAAA  Reverse : TGTCTGTTGGCTTCCTGGTCAG |
| *Ccl3* | Forward : ACTGCCTGCTGCTTCTCCTACA  Reverse : ATGACACCTGGCTGGGAGCAAA |
| *Ccl2* | Forward: TAAAAACCTGGATCGGAACCAAA  Reverse: GCATTAGCTTCAGATTTACGGGT |
| *Cd44* | Forward : CGGAACCACAGCCTCCTTTCAA  Reverse : TGCCATCCGTTCTGAAACCACG |
| *Csf1r* | Forward : TGGATGCCTGTGAATGGCTCTG  Reverse : GTGGGTGTCATTCCAAACCTGC |
| *Trem2* | Forward: CTGGAACCGTCACCATCACTC  Reverse: CGAAACTCGATGACTCCTCGG |
| *Marco* | Forward: CCTCCAGGGACTTACGGGT  Reverse: CCAGTGAGACCTATGTCACCT |
| *Krt7* | Forward: CGGAGATGAACCGCTCTATCCA  Reverse: CATGAGCATCCTTGATTGCCAGC |
| *F4/80* | Forward: CTGCACCTGTAAACGAGGCTT  Reverse: GCAGACTGAGTTAGGACCACAA |
| *hMETTL3* | Forward: CTATCTCCTGGCACTCGCAAGA  Reverse: GCTTGAACCGTGCAACCACATC |
| *hMIF* | Forward: AGAACCGCTCCTACAGCAAGCT  Reverse: GGAGTTGTTCCAGCCCACATTG |
| *hCSF1* | Forward: TGAGACACCTCTCCAGTTGCTG  Reverse: GCAATCAGGCTTGGTCACCACA |
| *hSAA1* | Forward: TCGTTCCTTGGCGAGGCTTTTG  Reverse: AGGTCCCCTTTTGGCAGCATCA |
| *hSAA2* | Forward: TCGTTCCTTGGCGAGGCTTTTG  Reverse: AGGTCCCCTTTTGGCAGCATCA |
| *h18S* | Forward: CGTCGGAAAACATCAGCCTCGT  Reverse: GAACGAGCTGTGTCTGGAAGAG |
| *mMif*  (for meRIP) | Forward: AGAGGGGTTTCTGTCGGAGC  Reverse: TAGTTGATGTAGACCCGGTCC |
| *mCsf1*  (for meRIP) | Forward: GTCTTGCTGACTGTTGGGGG  Reverse: GCTGCCCTTCCTCCAATGCA |
| *hMIF*  (for meRIP) | Forward: AGCCGGACAGGGTCTACAT  Reverse: ACGCTGTGTTCTAGGCCCGC |
| *hCsf1*  (for meRIP) | Forward: GCTGCCCTTCCTCCAATGCA  Reverse: GCAAGGCGGTACCAGTTACA |
| *NT* (for meRIP) | Forward：AGCGAAGGTGTGCTTTGAGA  Reverse：GAGGGTCAATGGCGTTCTGA |

NT: A flank region with no signal was used as a negative control.

**Table S3. Antibodies used in this study.**

| Name | Target | Host | Supplier | Cat no. | Application |
| --- | --- | --- | --- | --- | --- |
| α-SMA | Human, Mouse, Rat, Hamster, Monkey | Rabbit | Cell Signaling Technology | 19245 | IF, IHC,  WB |
| Col1a1 | Human, Mouse, Rat, Monkey | Rabbit | Cell Signaling Technology | 72026 | IF, IHC, WB |
| Krt19 | mouse | Rabbit | Abcam | 133496 | IF, IHC, Muti-IHC, WB |
| Ki67 | Human, Mouse, Rat | Rabbit | Cell Signaling Technology | 9129 | IHC |
| Ecad | Human, Mouse | Goat | R&D | AF748 | IF |
| F4/80 | Mouse | Rabbit | Cell Signaling Technology | 70076 | IF, IHC, Muti-IHC |
| Trem2 | Mouse, Rat | Rabbit | Bioss | bs-2723R | IF |
| Spp1 | Mouse | Rabbit | abcam | 218237 | Muti-IHC |
| CD44 | Human, Mouse, Rat | Rabbit | Cell Signaling Technology | 37259 | Muti-IHC |
| Krt7 | Human，Mouse, Rat | Rabbit | Abcam | 181598 | IF, IHC |
| METTL3 | Human，Mouse, Rat | Rabbit | Abcam | 195352 | Muti-IHC,  WB |
| Glul | Human，Mouse, Rat | Rabbit | Abcam | 176562 | IF |
| CD31 | Human，Mouse, Pig | Rabbit | Abcam | 28364 | IHC |
| CD163 | Human，Mouse, Rat, | Rabbit | Abcam | 182422 | IHC |
| Marco | Mouse | Rabbit | Abcam | 239369 | IF, IHC |
| Pdgfβ | Human, Mouse, Rat | Rabbit | Cell Signaling | 3169S | WB |
| GAPDH | Human, Mouse, Rat, Monkey | Rabbit | Cell Signaling Technology | 8884 | WB |
| Anti-Rabbit | - | Goat | ZSGB-bio | PV-6001 | - |
| MIF | Human | Mouse | R&D systems | MAB289 | Neutralizing Antibody |
| CSF1 | Human | Rabbit | Abcam | ab9693 | Neutralizing Antibody |
| Alexa Fluor^TM^ 488 donkey anti -goat IgG(H+L) | - | Donkey | Invitrogen | A11055 | - |
| Alexa Fluor^TM 568^ donkey anti -rabbit IgG(H+L) | - | Donkey | Invitrogen | A10042 | - |

IF: Immunofluorescence; IHC: Immunohistochemistry; Multi-IHC: Multi-color Immunohistochemistry; WB: Western blot.
